# Global Shipping Emissions from a Well-to-Wake Perspective: The MariTEAM Model

**DOI:** 10.1021/acs.est.1c03937

**Published:** 2021-10-27

**Authors:** Diogo Kramel, Helene Muri, YoungRong Kim, Radek Lonka, Jørgen B. Nielsen, Anna L. Ringvold, Evert A. Bouman, Sverre Steen, Anders H. Strømman

**Affiliations:** † Industrial Ecology Programme, NTNU, Trondheim 7034, Norway; ‡ Department of Marine Technology, NTNU, Trondheim 7052, Norway; § SINTEF Ocean AS, Trondheim 7052, Norway

**Keywords:** Shipping, Emissions, Life-cycle assessment, Decarbonization

## Abstract

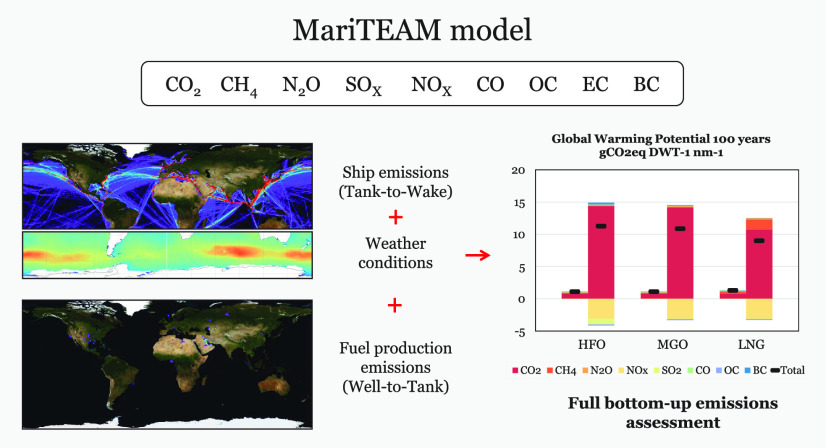

Improving the robustness of maritime emission inventories is important to ensure we fully understand the point of embarkment for transformation pathways of the sector toward the 1.5 and 2°C targets. A bottom-up assessment of emissions of greenhouse gases and aerosols from the maritime sector is presented, accounting for the emissions from fuel production and processing, resulting in a complete “well-to-wake” geospatial inventory. This high-resolution inventory is developed through the use of the state-of-the-art data-driven MariTEAM model, which combines ship technical specifications, ship location data, and historical weather data. The CO_2_ emissions for 2017 amount to 943 million tonnes, which is 11% lower than the fourth International Maritime Organization’s greenhouse gas study for the same year, while larger discrepancies have been found across ship segments. If fuel production is accounted for when developing shipping inventories, total CO_2_ emissions reported could increase by 11%. In addition to fuel production, effects of weather and heavy traffic regions were found to significantly impact emissions at global and regional levels. The global annual efficiency for different fuels and ship segments in approximated operational conditions were also investigated, indicating the need for more holistic metrics than current ones when seeking appropriate solutions aiming at reducing emissions.

## Abstract 2

This work presents a new model for historical emissions from global shipping and highlights the importance of using a well-to-wake approach.

## Introduction

As a consequence of the increasing demand for maritime transportation, emissions could increase proportionally. Despite being one of the most efficient freight modal options in terms of emissions per tonnage transported per kilometer,^1^ estimations by Faber et al.^2^ indicate that the sector could be responsible for around 2.9% of total anthropogenic carbon dioxide (CO_2_) emissions, 11% of sulfur oxides (SO_X_), and 15% of nitrogen oxides (NO_X_) in 2018. If other sectors, for example, agriculture or energy, were to adopt more stringent mitigation policies, the relative contribution to total anthropogenic emissions from the maritime sector could increase substantially unless action is taken. The International Maritime Organization (IMO) has adopted a strategy for reducing greenhouse gases (GHG). The current ambitions involve cutting emissions by at least 50% by 2050 compared to 2008, while, at the same time, pursuing efforts toward zero emissions by 2100 if not sooner.^3^ Additionally, strategies to mitigate other emissions (NO_X_, SO_X_, black carbon) are also being adopted (see, e.g., ref (4)).

For monitoring the progress of mitigation efforts, detailed emission inventories (EIs) that present the amount of various pollutants emitted spatially across the globe are necessary. Different inventory approaches have been developed for the maritime sector, but results are not always in agreement. For instance, the total amount of CO_2_ emitted by international shipping might vary across the years between ∼700 and ∼1300 million tonnes depending on the study^5−9,2,10^ and the approach, i.e., bottom-up or top-down. While top-down approaches generally lack a consistent geospatial representation and are believed to underestimate emissions, considering they are based on bunker fuel sales, the so-called bottom-up approach has been considered more representative as it models the emissions at individual ship level at a specific location. Such studies include the IMO GHG studies^9,2^ and the Ship Traffic Emission Assessment Model (STEAM) model.^10^

A comparison of the formulations behind such models was done by Nunes et al.^11^ and Moreno-Gutiérrez et al.^12^ followed by a study showing that different models could differ up to 40% in ship emissions.^13^ In fact Psaraftis and Kontovas^14^ pointed out the high number of assumptions in the IMO GHG studies and the lack of transparency. One of the main concerns is the choice of ship power calculation method, where the simpler Admiralty coefficient, based on adjusting the engine operation to a reference value (load-based), could yield significantly higher emission numbers than more modern methods, based on power prediction models (resistance based),^15^ applied in this study. This points to the need for a transparent modeling approach with a comprehensive level of detail and a profound understanding of the sector and maritime engineering when establishing emission inventories.

While it is evident that increased scientific consensus on the emissions from shipping is advantageous, it is essential to acknowledge that this does not imply the need for a consensus on one single calculation approach or model. Independent experiments and models are vital to establishing the degrees of agreement and confidence in scientific results. There is a long tradition for intercomparison exercises in the Earth system and integrated assessment modeling, exemplified by the Coupled Model Intercomparison Project (CMIP)^16^ and the Stanford Energy Modeling Forum (EMF).^17^ A model intercomparison approach could also be adopted for sectoral models to help build consensus on emissions from the shipping sector.

Furthermore, most studies focus on the direct emissions from the engine during ship operation and do not include emissions during other phases of the life cycle. This can be especially important for emissions of aerosols and particulate matter as these may occur nearer to urban settlements and contribute to air pollution and related human health hazards.^4^ Analyzing the emissions for the entire life cycle has been undertaken by Bengtsson et al.,^18^ Brynolf et al.,^19^ Chatzinikolaou and Ventikos, and Corbett and Winebrake,^21^ who developed models to analyze the emission of individual ships but has not been integrated into EIs yet in a global assessment.

The inclusion of the effects of wind and waves in ship performance, robust engine emissions models, and fuel production emissions while assessing ship emissions is an inherently complex problem, although essential to build appropriate regulations to curb climate change. Therefore, this study aims first to establish a new high-resolution data-driven emission inventory for the maritime sector, then compare this to existing emission inventories addressing differences in the life-cycle emissions and detail the importance of different model features in affecting the emissions globally and regionally in a transparent way.

## Materials and Methods

**Figure 1:**
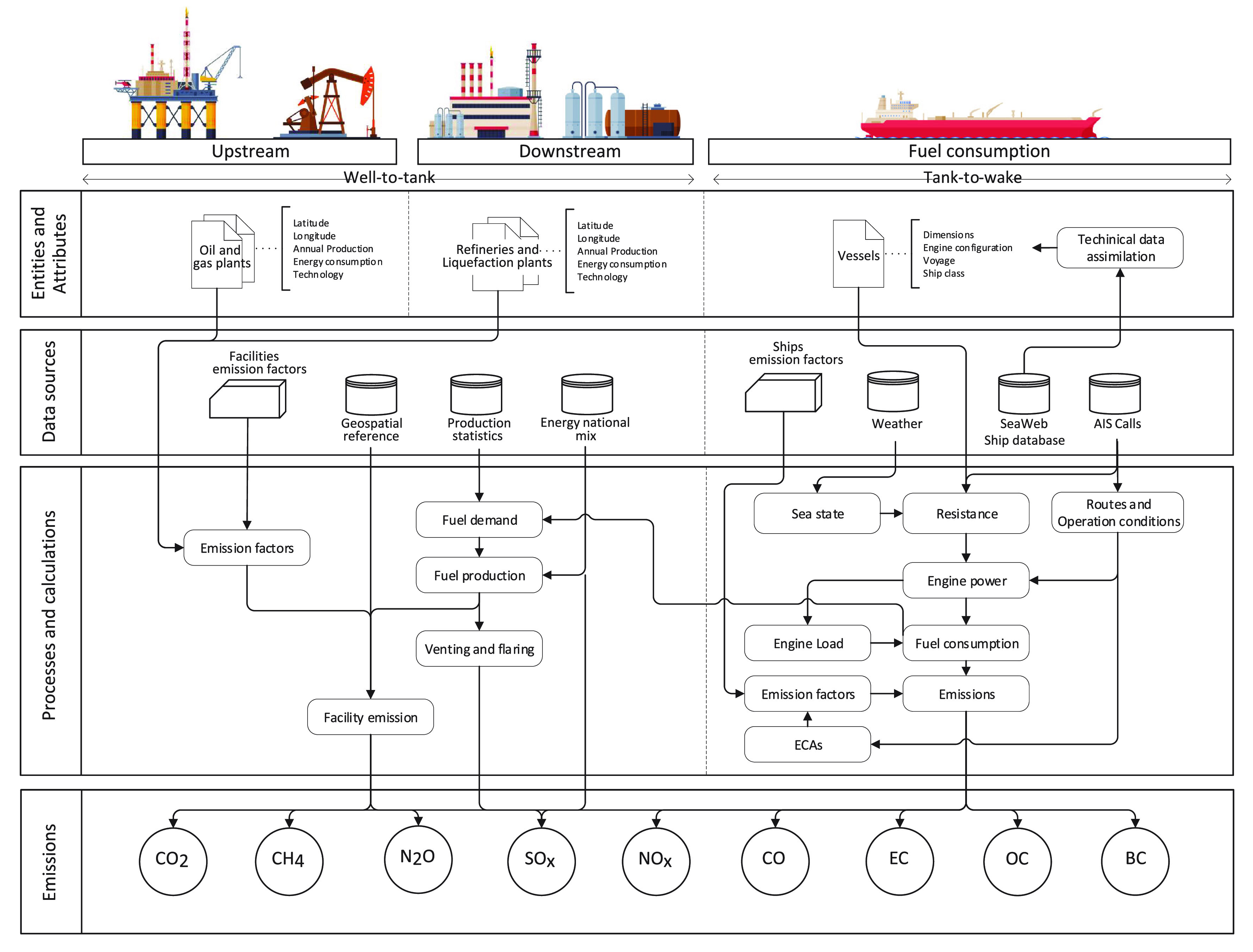
MariTEAM modeling framework for global well-to-wake emissions that combines different data sources to create virtual entities that represent the most important processes in the calculation of atmospheric emissions.

The global shipping emission assessment in this study was carried out following the framework in Figure 1 with the MariTEAM Model (**Mari**time **T**ransport **E**nvironmental **A**ssessment **M**odel) ship emission calculations at its core. These processes are described in detail in the following sections and in depth in the Supporting Information.

The model performs a life-cycle assessment (LCA) that includes the most emission-intensive phases of fuel production (well-to-tank), i.e., raw material extraction and its transportation and processing, as well as its combustion as direct ship emissions (tank-to-wake). In each process, emissions are modeled either as stationary points, corresponding to the facilities for fuel production, and nonstationary emission points, in which ships are individually characterized in the model. Each of these elements has specific emission modules that are dependent on the technology, vintage, and location, among others. The results are given as geospatial explicit emissions for CO_2_, CH_4_, N_2_O, NMVOC (nonmethane volatile organic compounds), SO_2_, SO_4_, CO, OC (organic carbon), EC (elemental carbon), and BC (black carbon) based on the global fuel mix, i.e., heavy fuel oil (HFO), marine gas oil (MGO), and liquefied natural gas (LNG). The quality of the fuel, impurities, and different chemical compositions are not taken into consideration, as well as the usage of fuel blends.

Direct ship emissions are the largest source of GHGs (greenhouse gases) in maritime transport, estimated to account for nearly 90% of life cycle emissions, depending on engine parameters and fuel properties.^22^ Contrary to fuel production, the emissions at this phase are characterized by being highly mobile and spread unevenly across the globe. Therefore, to represent them temporally and spatially in a high-resolution dimension, ships are modeled individually based on extensive data collection and processing.

### Technical Information on Ships

Ship technical parameters are organized in a database developed on the basis of the Sea-web Ships database that contains more than 200,000 ships of 100 gross tonnage (GT) and above, from which we have included only IMO-registered vessels that effectively operated in the year 2017, totaling 45,891 vessels. In terms of deadweight tonnage, it is 5.8% larger than the fleet covered by Faber et al.^2^ for the ship segments under analysis, while differences for segments are not greater than 4.2%, which is detailed in the Supporting Information (S1).

The ship characteristics database is preprocessed, and the missing ship parameters are filled based on a novel approach introduced by Kim et al.^23^ The method applies multiple linear regression models developed for each ship class, which accounts for the differences in ship characteristics among ship types obtaining an adjusted R^2^ in the range of 0.89–0.99. For instance, the most common parameters that have been filled for container ships are the auxiliary engine power (38.0%), light displacement tonnage (27.4%), and main engine’s RPM (2.5%), which are not expected to have an impact in the power prediction models applied.

For the spatial and temporal displacement of vessels, the sailing routes are obtained through a collection of terrestrial AIS (Automated Identification System) messages, combined with data obtained by two satellites: NorSat-1 and NorSat-2 launched in 2017 by NSC (Norwegian Space Center) in cooperation with Kystverket (Norwegian Coastal Administration). The NorSat-satellites alone collect approximately 1.5 million messages from about 50,000 ships per day.^24^ In this study ∼487 million AIS messages were used, after cleaning the data set to remove erroneous data points; i.e., data points on land were either reallocated to nearest waters if close by or disregarded. Port callings obtained from IHS Markit were included, with data on date and time of arrival and departure from ports for each vessel. For regions where the coverage was lacking and there was a larger gap in between the AIS messages, the routes were completed for shorter time intervals (0.1° latitude–longitude resolution) using the A* path-search algorithm^25^ in combination with Dijkstra’s algorithm.^26^ A demonstration of these method capabilities for completing voyages is shown in the Supporting Information (S2). The number of completed location points corresponds to an addition of ∼286 million, which accounts for 37% of the total ship location points used. The ship characteristics and routes were assigned to each vessel, and the combination of these two data sets made it possible to calculate the power demand at a ship level.

### Ship Resistance

Different methods have been proposed to estimate ship powering at a fleet-wide scale. Among them, Kristensen and Lutzen^27^ combined the ITTC-1957 model-ship correlation line and Harvald’s^28^ method. Lindstad et al.^29^ introduced an empirical model and a subsequent extension^30^ that includes the effect of weather for different sea states. Jalkanen et al.^31^ made use of Hollenbach’s^32^ method for calm water resistance and Kwon’s^33^ method for added resistance. The IMO GHG studies opt for using the Admiralty formula (for details, see, e.g., Schneekluth and Bertram^34^) adjusted with the Holtrop and Mennen^35^ method, plus a percentual sea margin depending on ship type to estimate the ship’s instantaneous required propulsive power based on its instantaneous speed and current draft. The main conceptual difference in IMO’s approach is that load-factor-based model adjusts the propulsive power for a reference point, scaling it based on the change in instantaneous speed and draft at the power of 3 and 0.66, respectively, while Schneekluth and Bertram^34^ have shown these values could vary for different ship types, which can lead to significant differences as it moves further away from the reference point.

For this reason, in the MariTEAM model, we adopt a resistance-based approach to calculate the aerohydrodynamic forces, from which the instantaneous power demand can be derived, using a combination of different methods. The frictional component of the resistance calculation is based on the ITTC-1957 model-ship correlation line, using an empirical method proposed by MARINTEK for the form factor (*k*). For the residual resistance, which corresponds to the energy loss caused by waves created by the vessel, we combine the methods of Holtrop and Mennen^35^ and Hollenbach^32^ widely used for ship emission estimations.^11^ For the resistance component originating from the effect of wind speed and direction, the approach used combines the methods of Blendermann^36^ and STAJIP.^37^ For the added resistance due to waves, we apply the STAWAVE-1 method and complement it with the STAWAVE-2 method^38^ that approximates the transfer function of the mean resistance increase in heading regular waves. A more detailed description is given in the Supporting Information (S3).

The historical meteorological conditions, from which the wind and sea state parameters are obtained, are acquired from the ERA-Interim (ECMWF Re-Analysis) data set provided by the European Centre for Medium-Range Weather Forecasts (ECMWF). Shi et al.^39^ observed in their measurements that significant wave heights measured by buoys differ by ±0.09 m when compared with ERA5, the latest version of the ERA reanalysis data sets. The wind and ocean wave data set contains data in 6h time intervals at a 1.0° latitude and longitude grid resolution^40^ and are assigned to ships as weather states throughout the year, depending on time and location.

### Ship Propulsion

The instantaneous power demanded at the propeller is a direct function of the total hull resistance (*R*_T_) and the ship’s velocity at a given point in time (*V*_S_). To obtain the power delivered by the engine, we have to account for the open-water efficiency (η_0_), hull efficiency (η_H_), relative rotative efficiency (η_R_), and transmission efficiency (η_S_), as shown in eq 1 and detailed in the Supporting Information (S4).
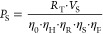
1

Another factor included is the effect of hull and propeller fouling increase throughout the years represented by an additional coefficient (η_F_) that may increase ship resistance to an estimated rate of 2%–11%.^41^ When applied across the global fleet, ship resistance increases on average by 6% with different results depending on ship type, in comparison to IMO GHG study’s fouling factor of 9%, This is detailed in the Supporting Information (S5). This is included in the model assuming ships are cleaned and have a new coating applied every 5 years based on values suggested by Lu et al.^42^ For auxiliary engines and boilers, the modeling process is detailed in the Supporting Information (S6). The ship propulsion formulation applied in this study has been tested against ship in-service data, as shown in the Supporting Information (S7) for one of the vessels analyzed.

### Ship Emissions

For calculating the amount of emissions, two methods are adopted depending on the pollutant. The first method is based on the amount of fuel consumed and its chemical composition, i.e., carbon and sulfur content. The second method aims to measure the emission species that are more likely to be affected by main engine parameters and the combustion process, which have been developed through regression models based on on- and off-board measurements, i.e., CH_4_, N_2_O, NMVOC, CO, OC, EC, and BC.

Both methods make use of emission factors that correlate the fuel or energy consumption with the total emissions. The emission factors are subject to the engine parameters (engine vintage, RPM, number of strokes, installed power) and engine load throughout its operation based on the maximum continuous rating (MCR) and are hence referred to as emission curves. Additionally, emissions are directly affected by emission restrictions in ECAs (Emission Control Areas) that limit the sulfur content in fuel and NO_X_ emissions. More details are found in the Supporting Information (S8).

### Fuel Production Emissions

Fuel production emissions are modeled as stationary sources covering the extraction, production, processing, storage, and transportation of each fuel analyzed, i.e., HFO, MGO and LNG, and their annual production is designed to meet global energy needs of fuel for the maritime sector, evenly distributed according to their production capacity. Furthermore, facilities are distinguished by specific emission factors (EF) dependent on installed technologies, location, and vintage. The facilities also include the calculation of emissions due to the electricity demand (EM) in its processes, which is based on each facility’s national electricity mix (EF_EM_). Losses due to material waste, leakage, venting, and flaring are also included. Methane leakage throughout the LNG supply chain (including pipeline transportation) was evenly distributed across wells and liquefaction plants. The material flow analysis (MFA) applied in this study is shown in the Supporting Information (S9). Equation 2 summarizes the calculation for a generic facility.
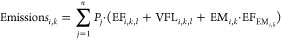
2

The coefficient *j* corresponds to the facility, *k* the spatial location (latitude and longitude), *i* the emission species, and *l* a set of technical characteristics for each facility.

The upstream production covers the extraction of crude oil and natural gas from hydrocarbons fields onshore and offshore and its processing. Oil and gas reserves vary widely in terms of reservoir accessibility, well properties, and hydrocarbon quality. For instance, the average carbon intensity (CI) in Venezuela is estimated to be 4 times higher than in Saudi Arabia.^44^ For this reason, emission factors specific for macroregions from the 2016 Environmental Performance Indicators,^43^ i.e., Africa, Asia/Australasia, Europe, Middle-East, North America, Russia and Central Asia, and South and Central America, have been combined with the studies of Masnadi et al.^44^ and the ICCT’s report by Malins et al.^45^ The flaring rate, venting, and losses in extraction have been modeled at region level based on the 2016 Environmental Performance Indicators.^43^

Regarding the location, several databases have been combined to obtain the facilities geospatial distribution and properties, covering 83% of crude oil extraction facilities and 78% for natural gas in terms of reported annual production. The emissions related to fuel transportation from production facilities to storage tanks are included as ship emissions when fuel is transported by oil and liquefied gas tankers or they are aggregate with fuel production facilities when transported by pipelines.^46^

After extraction, crude oil is processed in refineries to produce distillate low-sulfur fuel oils (MGO) and other byproducts, such as heavier oils (HFO), while natural gas is processed in liquefaction plants to remove impurities. The emissions occurring at the production of fuel oils are modeled based on the study of Jing et al.^47^ that provides country-level emissions based on the Oil Production Greenhouse Gas Emissions Estimator (OPGEE) model^48^ and the Petroleum Refinery Life Cycle Inventory Model (PRELIM) model.^49^ Similar to oil and gas fields, the fuel processing facilities are combined with spatial information, covering nearly 86% of all existing refineries in annual processing capacity. For natural gas, 98% of all liquefaction plants are covered in this study.^50^ This process, due to similarities with the oil production life cycle, is modeled in a similar manner. In both cases, it is not possible to track the supply chain and determine how each hydrocarbon field contributes to the production of different fuels; therefore, fuel demand is distributed to each field proportionally to its annual production capacity.

## Results and Discussion

In this section, we present the well-to-wake emissions for the MariTEAM model based on 2017 activity, followed by a comparison with other bottom-up assessments. The fuel consumption for that year included the usage of HFO (79% of total fuel), MGO and low-sulfur distillate fuels (18%), and LNG (3%).^2^ Methanol was not included due to its near-negligible usage.

### Geospatial Distribution

**Figure 2:**
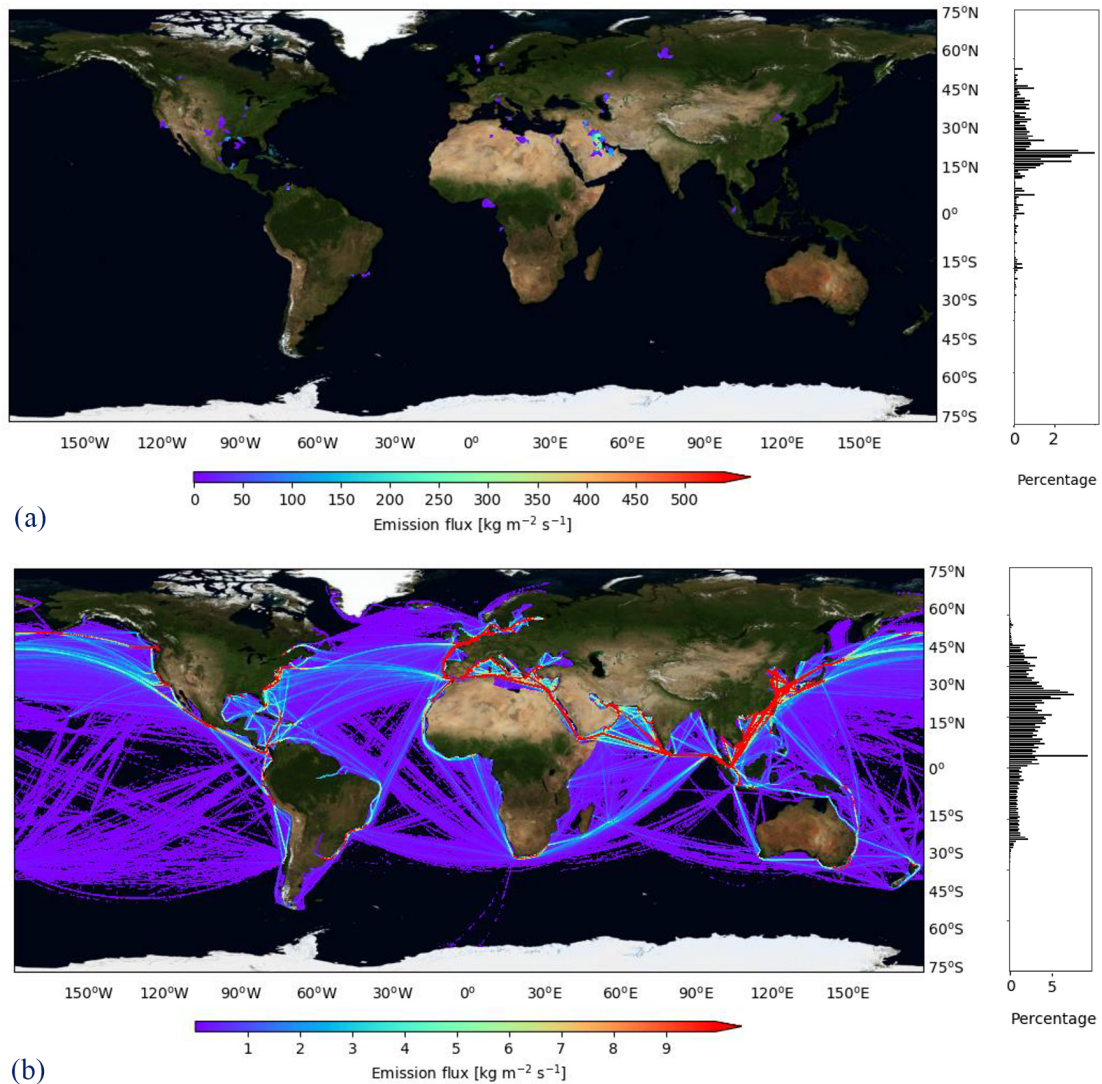
Geospatial distribution of CO_2_ emissions (kg m^–2^ s^–1^) for well-to-tank (a) and tank-to-wake (b) global shipping in the year 2017 with percentual latitude distribution (%).

The emissions have been calculated geospatially based on the positions of ships and fuel production facilities and summed in each 0.1° grid box over the full year (Figure 2). For fuel production (Figure 2a), the major contribution originates in the Persian Gulf (35% of total emissions), where the highest production oil fields are located. This is a significant difference to other hotspots, such as the Gulf of Mexico (2.9%) and the North Sea (3.3%).

For fuel processing, several facilities are distributed across Europe and in North America. Many of these facilities are located inland and are not exactly representative to where distillate fuel and LNG are processed for maritime fuels. The scale of emission is, naturally, higher than ship emissions since the points are stationary.

For ship emissions (Figure 2b), the regions with the highest ship emissions per square area are found across the Mediterranean, in the European Coast, and Southern-East Asia, which are typically identified as the shipping lanes with the most traffic.^10^ The geospatial distribution shows a major concentration near the Malacca Strait that helps to bring ship emissions to 87% in the Northern Hemisphere. Maps of emission fluxes of all species calculated can be found in the Supporting Information (S10 and S11).

### Emissions Inventory Results and Comparison with Previous Studies

The most recent studies to assess emissions for international and global shipping have been published by Johansson et al.^10^ that estimated 831 million tonnes of CO_2_ for the year 2015 (no weather effect included) and Faber et al.^2^ that calculated 1064 million tonnes for the year 2017. For the 2017 scenario, the MariTEAM model resulted in 943 million tonnes for global shipping. Of course, emissions for different years and scopes are not comparable. However, our total CO_2_ results being 2.3% larger than Johansson et al.^10^ (resistance-based model) adjusted to a sea margin of 12.5%, while 9.6% lower than the fourth IMO GHG (load-based model) study, indicate that different ship propulsion methods could directly affect the amount of emissions. The methodological differences in those and other previous studies, including bottom-up (BU) and top-down (TD) assessments, are illustrated in Table 1 that summarizes the main features in the modeling process and their results, indicating that the increase of robustness in the model could promote a convergence in CO_2_ emissions.

**Table 1:** Synthesis of Key Studies Covering Global Ship Emission Inventories, Including This Study and Bottom-Up and Top-Down Assessments, Indicating Features Present (●) or Absent (○) in Each Study

Features present in the study		(5)	(6)	(7)	(51)	(8)	(9)	(10)	(56)	(2)	This study
Fuels investigated	HFO	●	●	●	●	●	●	●	●	●	●
	MGO	●	●	●	●	●	●	●	●	●	●
	LNG	○	○	○	○	○	○	○	○	●	●
Pollutants	CO_2_	●	●	●	●	●	●	●	●	●	●
	CH_4_	○	○	●	●	○	●	○	○	●	●
	N_2_O	○	●	●	●	○	●	○	○	●	●
	NMVOC	○	●	●	●	○	●	○	●	●	●
	SO_X_	●	●	●	○	●	●	●	●	●	●
	NO_X_	●	●	●	○	●	●	●	●	●	●
	CO	○	●	●	●	○	●	●	●	●	●
	PM_10_	●	●	●	●	○	●	○	○	●	○
	BC	○	○	○	○	●	○	○	●	●	●
Ship emissions		BU	BU	BU	BU	BU	BU+TD	BU	BU	BU+TD	BU
Spatial distribution		○	TD	TD	TD	TD	○	BU	BU	BU	BU
Fuel production		○	○	○	○	○	○	○	○	○	●
AIS ship data		○	○	○	○	○	●	●	●	○	●
Weather modeling		○	○	○	○	○	○	●	○	○	●
Load curves		○	○	○	○	○	●	●	●	●	●
Hull coating		○	○	○	○	○	●	○	○	●	●
Number of vessels		88,660	87,546	90,363	32,000	40,055	45,041	76,000	69,399	104,608	45,891
Reference year		2001	2000	2001	2004	2006	2007–2012	2015	2015	2012–2018	2017
CO_2_ (10^6^ ton)		789	884	1306	689	695	938–1135	831	866	957–1064	943

We also compare the global annual emissions for 2017 with the fourth IMO GHG study (Table 2). Emissions of GHGs (CO_2_, CH_4_, N_2_O) are lower than what has been estimated by the IMO GHG study, with a difference of 11.4% for CO_2_ for tank-to-wake. Our emission inventory shows that accounting for emissions during fuel production and processing (well-to-tank) could increase total emissions (well-to-wake) between 12% (CO_2_) and 2% (CO), depending on the pollutant, when compared to standard ship emissions (tank-to-wake).

**Table 2:** Global Emissions in 2017: Tank-to-Wake and Well-to-Tank and Comparison between the MariTEAM Model and Fourth IMO GHG Study

			MariTEAM (Tank-to-wake)				MariTEAM (Well-to-tank)				Difference between MariTEAM and IMO	
Pollutant	Unit in tonnes	Fourth IMO GHG study	HFO	MGO	LNG	Total	HFO	MGO	LNG	Total	TtW	WtW
CO_2_	10^6^	1064	751	168	21	943	79	34	6	112	–11%	0%
CH_4_	10^3^	128.8	0.0	0.0	107	107	7.6	3.3	1.5	12	–17%	–7%
N_2_O	10^3^	59.4	40	9	1.5	50	2.6	1.2	0.1	3.9	–15%	–9%
NMVOC	10^3^	984	795	182	25	1001	28	12	10	42	2%	6%
SO_X_	10^6^	11.7	8.9	0.1	0.0	9	0.8	0.4	0.0	1.2	–23%	–13%
NO_X_	10^6^	23.2	15	3.4	0.5	19	1.5	0.6	0.1	2.2	–20%	–10%
CO	10^3^	955	505	123	14	642	9.7	4.3	0.4	15	–33%	–31%
OC	10^3^	–	136	31	5.2	173	–	–	–	–	–	–
EC	10^3^	–	12	2.6	0.1	15	–	–	–	–	–	–
BC	10^3^	99.7	23	1.9	0.0	25	1.4	0.6	0.1	2.1	–75%	–73%

When we compare our fuel production emissions with similar studies, they appear to agree with standard well-to-tank emissions. One of the most recent studies to assess the same fuels we have covered^52^ presents well-to-tank emissions for HFO of 9.6 gCO_2_eq/MJ (ours 9.4 gCO_2_eq/MJ), 14.4 for MGO (ours 13.9), and 18.5 for LNG (ours 17.9).

For tank-to-wake emissions, some pollutants are generally more difficult to quantify, and larger discrepancies between the EIs exist. Although Faber et al.^2^ opts to model PM and BC emissions, our study focuses on chemical species (OC, EC, BC). These can be defined differently between studies, and we follow the recommendations proposed by Petzold et al.^53^ in the stratification of black carbon measurements. This can explain the differences in BC emissions between EIs. This group of hydrocarbons, however, once summed present a difference of 18.1% compared to similar species in the IMO study.

**Figure 3:**
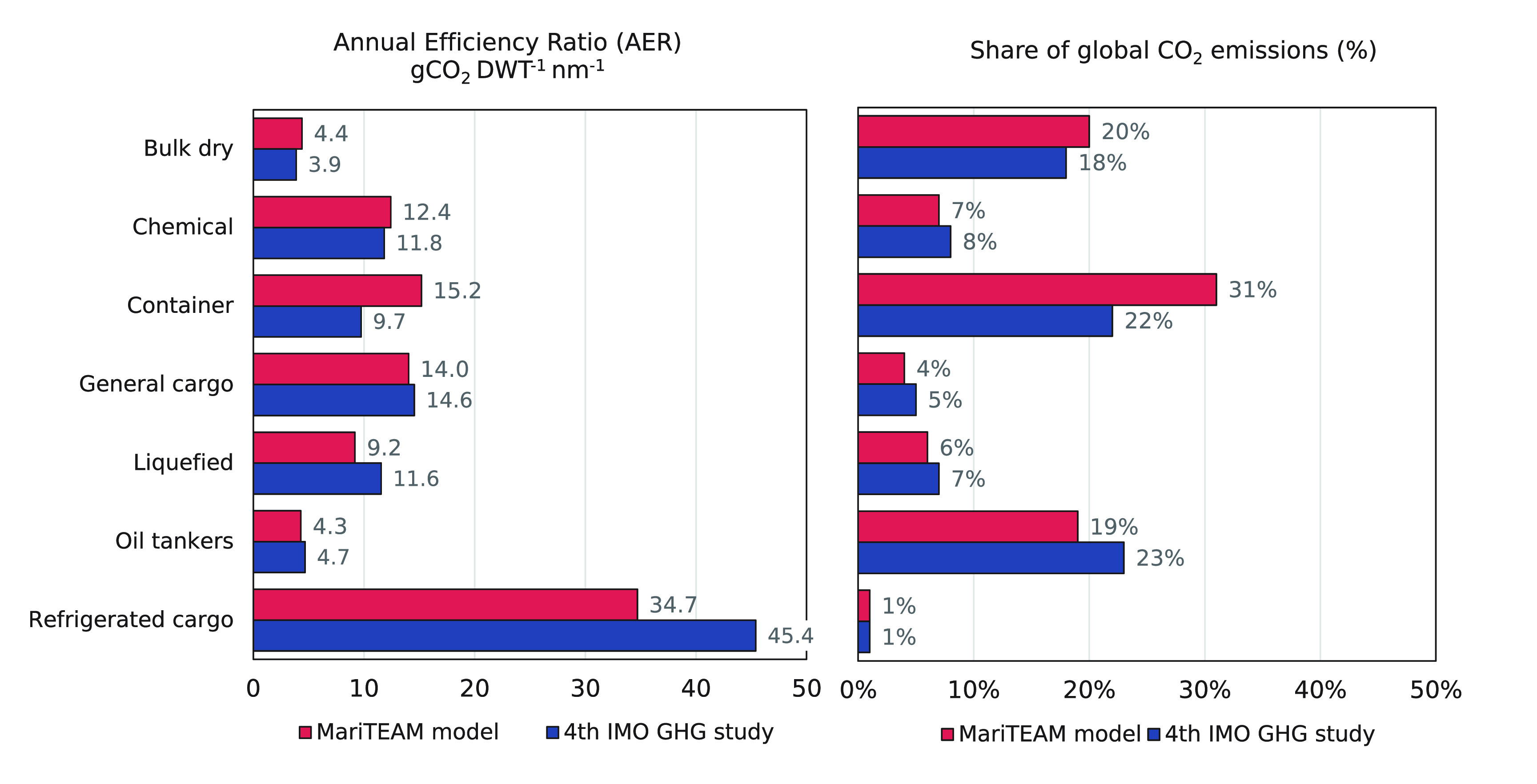
Annual efficiency ratio (AER) (gCO_2_ DWT^–1^ nm^–1^) for each ship type considered by the MariTEAM model (coral bar) and the fourth GHG study by the IMO (navy blue bar) and global CO_2_ contribution per ship type (same color scheme).

The CO_2_ emissions can also be aggregated by ship type to represent each segment’s global share (Figure 3). Emissions for oil tankers, bulk dry ships, and container ships account for 19%, 20%, and 31%, respectively, which differ from the fourth IMO GHG study (23%, 18%, 22%). These differences are reflected when combining emissions with transported cargo and distance sailed to obtain the annual efficiency ratio (AER).^54^

The lower values for container ships in Faber et al.^2^ could originate from the Admiralty Law that is not effective in capturing wave-making resistance, which is more significant for high-speed container ships than it is for tankers or bulk carriers. For all segments, the generally higher AER values in the MariTEAM model occur due to the total distance sailed that tends to be generally lower when compared with IMO’s results. Other differences between ship types are expected due to the sea margin applied in the IMO GHG studies.

**Figure 4:**
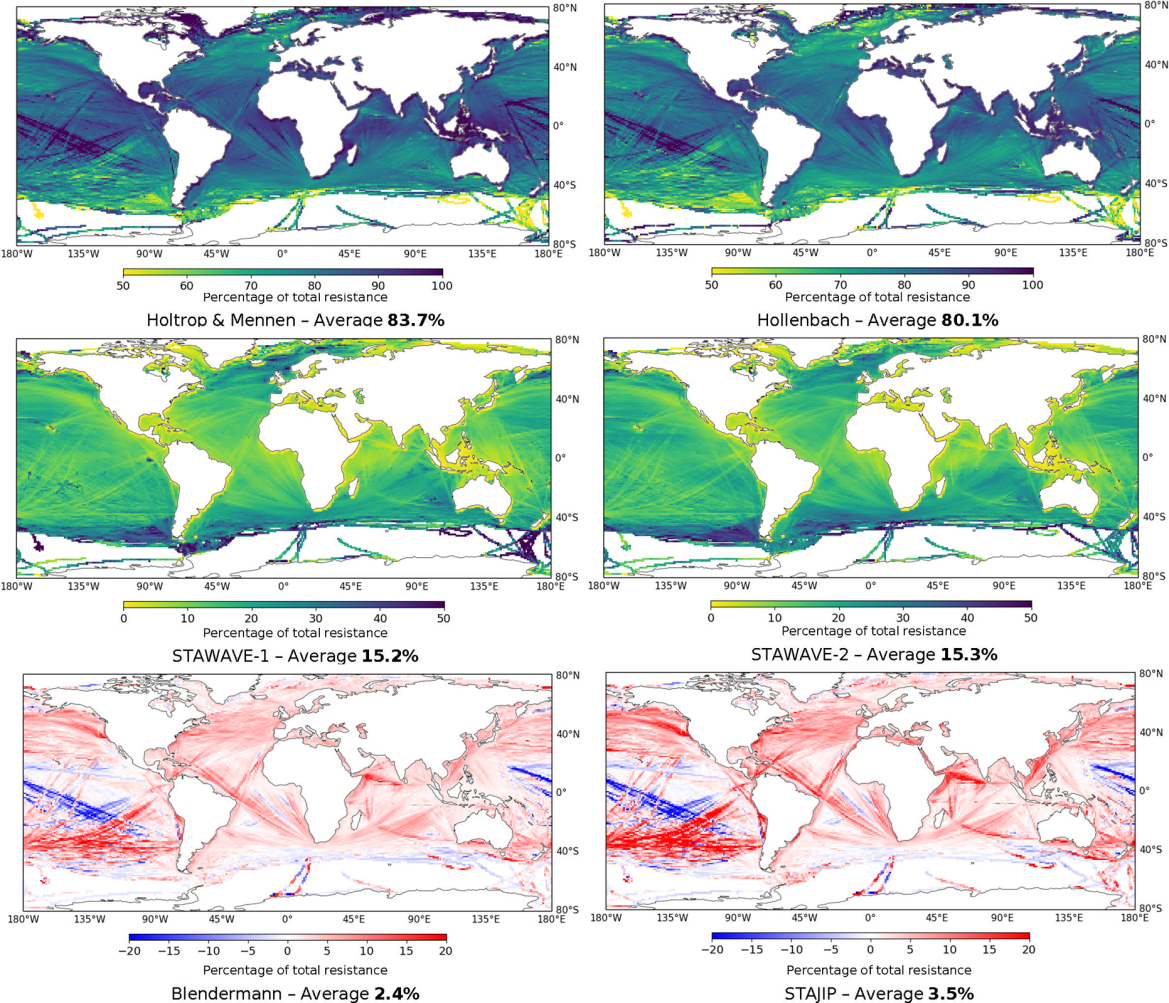
Average contribution to total ship resistance for calm water (top, i.e., Holtrop and Mennen and Hollenbach methods), added wave resistance (center, i.e., STAWAVE-1 and STAWAVE-2 methods), and added wind resistance (bottom, i.e., Blendermann and STAJIP method). The number in bold indicates the global mean value of the contribution to the total ship resistance.

In fact, the effect of the weather and the resistance models applied have shown to be crucial for assessing emissions in this study. Figure 4 shows how methods differ when compared to total average ship resistance calculated by the MariTEAM model.

For still water, Hollenbach’s method yields generally lower values (4.3%) than Holtrop and Mennen. For added resistance due to waves, both STAWAVE methods have similar average values, despite differences in their formulation. For added resistance due to wind, methods vary significantly, and STAJIP is in total almost 45% higher than Blendermann’s, which highlights the importance of combining different methods. The effect of wind can assume negative values as well in the case of stern wind.

The resistance components represent the global fleet average and can vary between different ship segments. For instance, for bulk dry ships, added resistance corresponds to 19% of total resistance from which only 1.3% corresponds to wind resistance, whereas for container ships that operate at higher speeds this contribution is 10% from which 3.8% is originated from wind, caused by the larger area above waterline. Assuming average factors across the fleet, as performed in other studies, could lead to underestimations in some segments and overestimations in others, affecting their annual efficiency ratio (AER) and potentially misdirecting policies targeted at specific segments.

Another factor that is important in this assessment is the suboptimal operation conditions that vessels undergo during voyage. For container ships and bulk dry carriers, the segments with the biggest shares in CO_2_ emissions, vessels spend ∼80% of their operation outside the 75%–90% MCR range. These values reinforce the aforementioned importance of adjusting emissions according to % MCR as crucial for increasing robustness of bottom-up emissions inventories.

### Climate Implications

The emission species arising from shipping activity can cause either a positive or negative contribution to radiative forcing on the climate. The lifetime can be relatively long for well-mixed GHG (longer than 100 years for CO_2_) or as short as days for SO_2_ and NO_X_. To assess their combined effect, the global warming potential (GWP) and global temperature potential (GTP)^55^ can be used. GWP considers the amount of energy a gas absorbs over a given period, whereas GTP evaluates the temperature changes at the end of the same period. These two metrics can be calculated through a combination of factors with our EI. The values applied in this study are based on Chapter 8 of the IPCC’s fifth assessment report (AR5)^1^ (Supporting Information, Table 8.SM.17).

**Figure 5:**
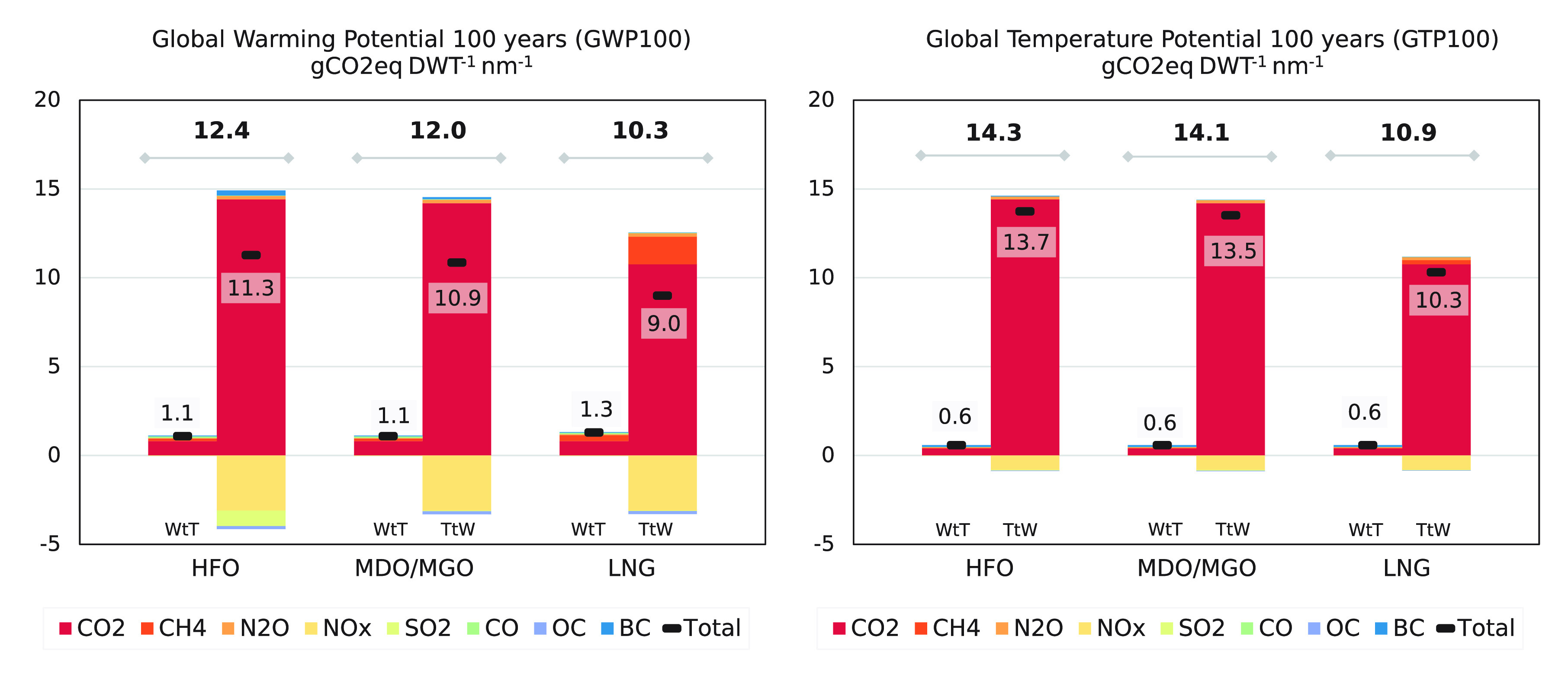
Contribution of emission species to GWP100 (a) and GTP100 (b) (gCO_2_eq DWT^–1^ nm^–1^) aggregated in well-to-tank and tank-to-wake emission for the three different fuels being analyzed, i.e., HFO, MGO, and LNG.

The results for a 1-year emission pulse are given as CO_2_ equivalent per transport work for both GWP100 and GTP100 to allow for a comparison between fuels with different global consumption (Figure 5). The cooling effects caused by NO_X_ and SO_X_ lead to a reduction in net forcing. However, with more stringent quality control through ECAs that significantly limit the permissible amount of SO_X_ and NO_X_ emissions, the cooling effects are expected to be lower in the future. For the specific case of natural gas, CH_4_ emissions in both fuel production and ship emissions offset in part the benefit of lower CO_2_ emissions, as methane’s GWP100 is approximately 28 times higher than CO_2_’s.

The contribution of fuel production increases the GWP100 by nearly 10% and 5% for GTP100. As shipping moves toward less carbon-intensive fuels (LNG) and potential carbon-free alternatives such as ammonia or hydrogen, well-to-tank emissions are expected to increase in absolute and relative figures, highlighting the importance of well-to-wake assessments and the importance of including fuel production emissions in bottom-up assessments.

Additionally, the discussion on using well-to-wake assessment may foster the development of new metrics to fully assess the emissions from maritime fuels. If the usage of low- or zero-carbon fuels increases, and therefore, direct operational ship emissions are dramatically reduced, the current 1:9 ratio between ship and upstream emissions would change. A holistic perspective for assessing emissions that include not only fuel production and ship emission but also other life-cycle phases neglected so far, such as emissions in port and during ship building and decommissioning, will become increasingly important in the future.

Thus far, emissions in the maritime sector have been assessed through different approaches that vary in their different underlying assumptions in terms of temporal–spatial resolution and how ships are individually modeled (e.g., bottom-up versus top-down), scope (e.g., ship-level versus global fleet), coverage (e.g., tank-to-wake versus well-to-tank), and how external factors (e.g., waves and wind) are included in the modeling process. Hence emission inventories are not always in consensus.

Although the emission inventory presented is in good agreement overall with the fourth IMO GHG study, we identify that the breakdown of emissions can substantially differ spatially, between segments, and operational conditions. Furthermore, the inclusion of fuel production may account for a non-negligible 11% increase in CO_2_ emissions, significant when considering alternative fuels that might result in even higher production emissions. In the face of that, there is a clear need for high resolution, full bottom-up models using a well-to-tank approach that are able to capture a variety of details and behavior within the sector to enable effective decarbonization.
