# Correction to “Global Shipping Emissions from a Well-to-Wake Perspective: The MariTEAM Model”

**DOI:** 10.1021/acs.est.2c01301

**Published:** 2022-04-19

**Authors:** Diogo Kramel, Helene Muri, YoungRong Kim, Radek Lonka, Jørgen B. Nielsen, Anna L. Ringvold, Evert A. Bouman, Sverre Steen, Anders H. Strømman

In Figure 3 in the original article, values for the oil tankers’ share of global CO_2_ emissions should read 15% for the MariTEAM model and 17% in the fourth IMO GHG study. Figure 3 with corrected values is shown here.

**Figure 3:**
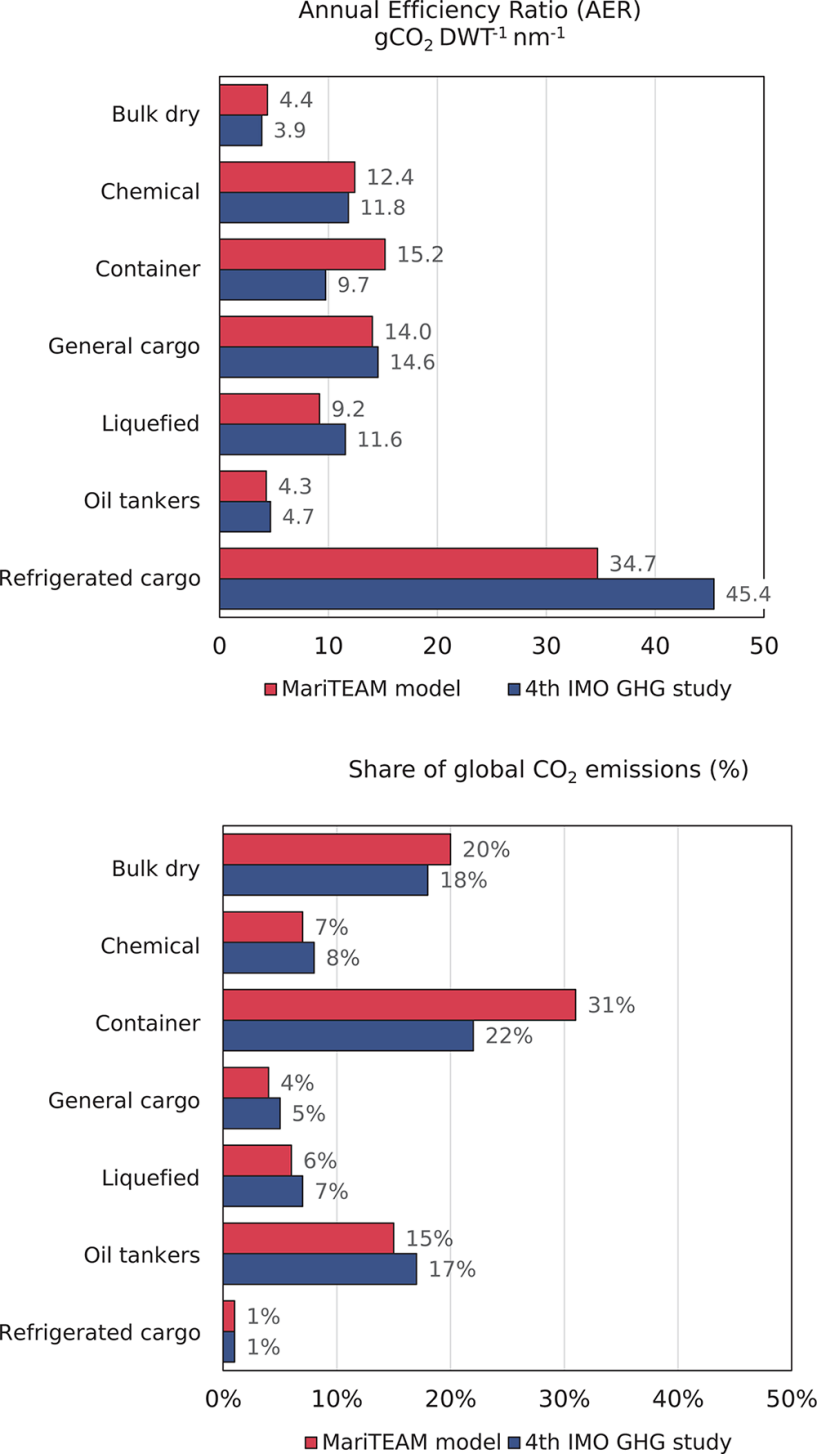
Annual efficiency ratio (AER) (gCO_2_ DWT^–1^ nm^–1^) for each ship type considered by both the MariTEAM model (coral bar) and the fourth GHG study by the IMO (navy blue bar) and global CO_2_ contribution per ship type (same color scheme).

Those values are to be applied to Figure S29 in the Supporting Information as well. As a consequence, the fourth paragraph of page 15046, should read as follows, with corrections highlighted in bold:

“The CO_2_ emissions can also be aggregated by ship type to represent each segment’s global share (Figure 3). Emissions for oil tankers, bulk dry ships, and container ships account for **15%**, 20%, and 31%, respectively, which differ from the fourth IMO GHG study (**17%**, 18%, 22%).”

Figure 5, the global warming and temperature potential for 100 years for the global fleet, with corrected values is shown here.

**Figure 5:**
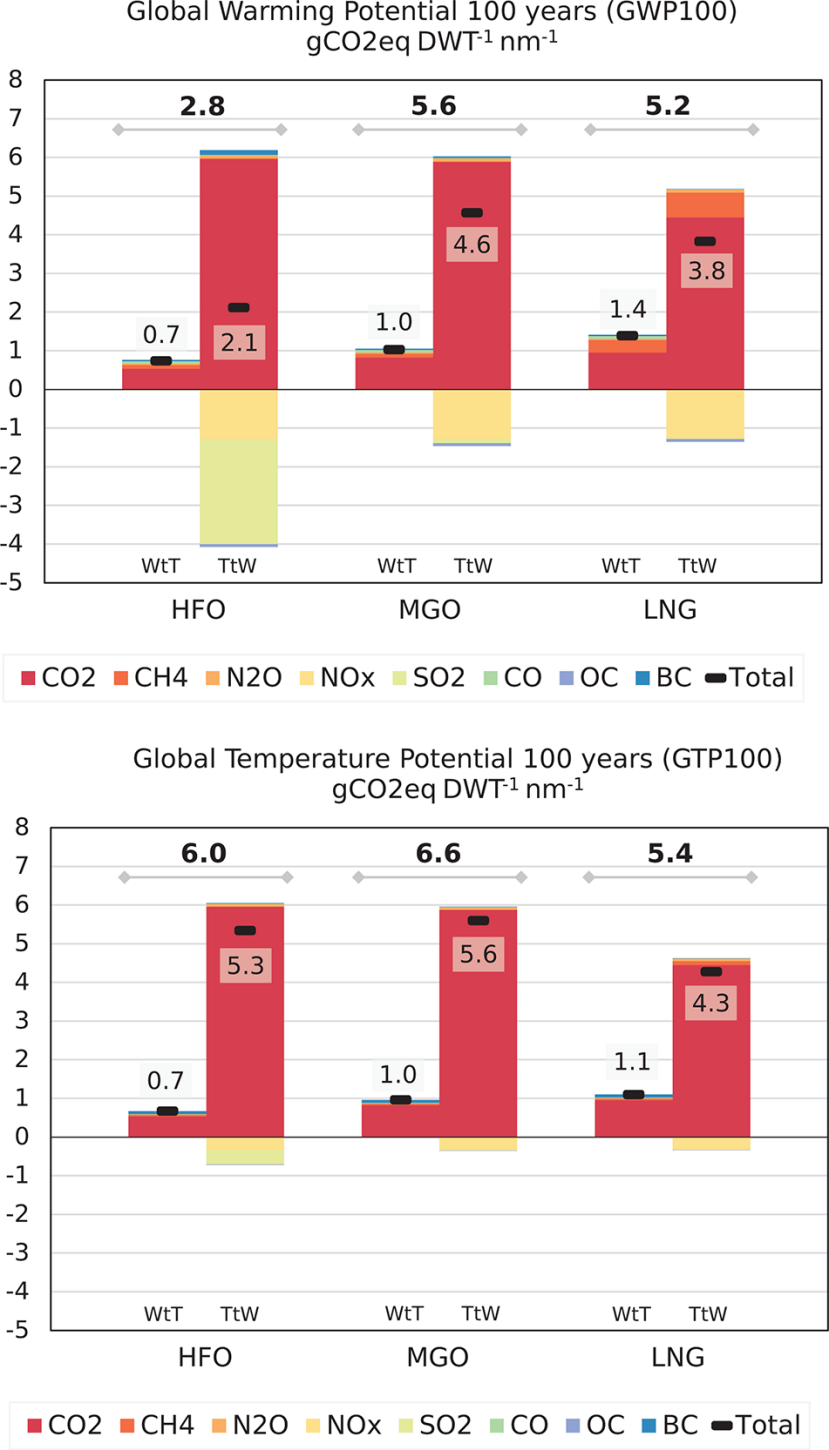
Contribution of emission species to GWP100 (a) and GTP100 (b) (gCO_2_eq DWT^–1^ nm^–1^) aggregated in well-to-tank and tank-to-wake emissions for the 2017 global fleet inventory based on data as provided in the Supporting Information, that is, HFO (2.6% sulfur content), MGO (0.1% sulfur content), and LNG (low-pressure dual-fuel (LPDF)).

On page 15047, the sixth paragraph therefore should read:

“The contribution of fuel production increases fleetwide GWP100 by nearly **33%** and **13%** for GTP100.”

Lastly, in the Acknowledgments section, individual contributions to the publication should read as follows:

“The authors also would like to thank Per Magne Einang and Anders Valland for the helpful suggestions, and also thank Mario Salgado Delgado for his contribution.”

The corrected Acknowledgments is shown here.

