# Supplementary material for: Global Shipping Emissions from a Well-to-Wake Perspective: The MariTEAM Model
Source: Environ Sci Technol. 2021 Oct 27;55(22):15040–50. doi: 10.1021/acs.est.1c03937 (PMC8600665; doi:10.1021/acs.est.1c03937)
Supplement: Supplementary file 1 — es1c03937_si_001.pdf [file es1c03937_si_001.pdf]

Supporting Information for:

Global shipping emissions from a well-to-wake perspective: the MariTEAM model

Diogo Kramel[,1], Helene Muri[1], YoungRong Kim[2], Radek Lonka[1], Jørgen B. Nielsen[3], Anna L. Ringvold[1], Evert A. Bouman[1,4], Sverre Steen[2], and Anders H. Strømman[1]*

[1] Industrial Ecology Programme, NTNU, Trondheim, Norway

[2] Department of Marine Technology, NTNU, Trondheim, Norway

[3] SINTEF Ocean AS, Trondheim, Norway

[4] Environmental Impacts & Sustainability, Norwegian Institute for Air Research (NILU) Kjeller, Norway

Text S1. Global fleet coverage

[*] diogo.kramel@ntnu.no

The fleet coverage in terms of numbers of vessels included in the MariTEAM model is compared to the number of vessels analysed in the 4th IMO GHG study. In Figures S1 to S5 we show bulk dry, chemical tanker, container, general cargo, and oil tankers. In addition, the MariTEAM model has been run with smaller segments, such as offshore supply, passenger, ro-ro, liquefied gas, refrigerated cargo, other offshore, other bulk, other cargo, and other liquids. This segmentation differs somewhat to the IMO GHG studies. The MariTEAM model include all vessels in the Sea-web Ships database that are IMO-registered and effectively operated in the year 2017, totalling 45891 vessels.

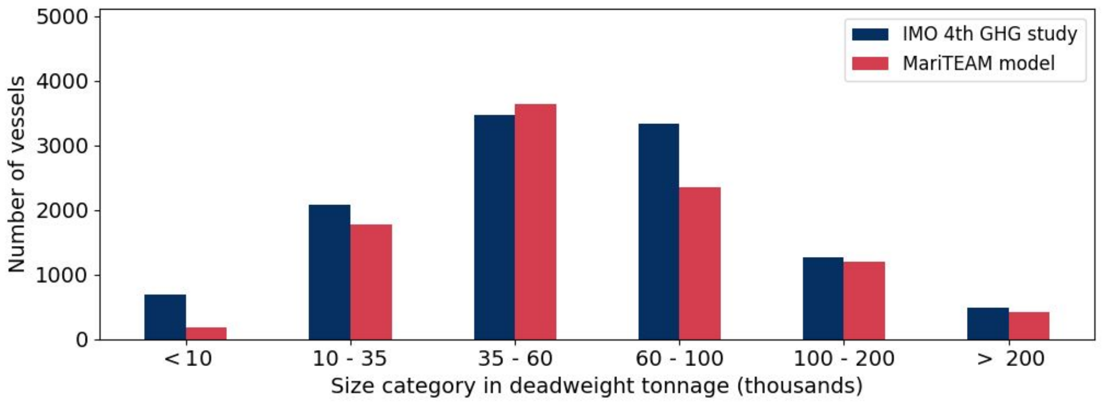

Figure S1. Comparison between number of bulk dry vessels binned by deadweight tonnage evaluated in the 4th IMO GHG study and with the MariTEAM model in this study.

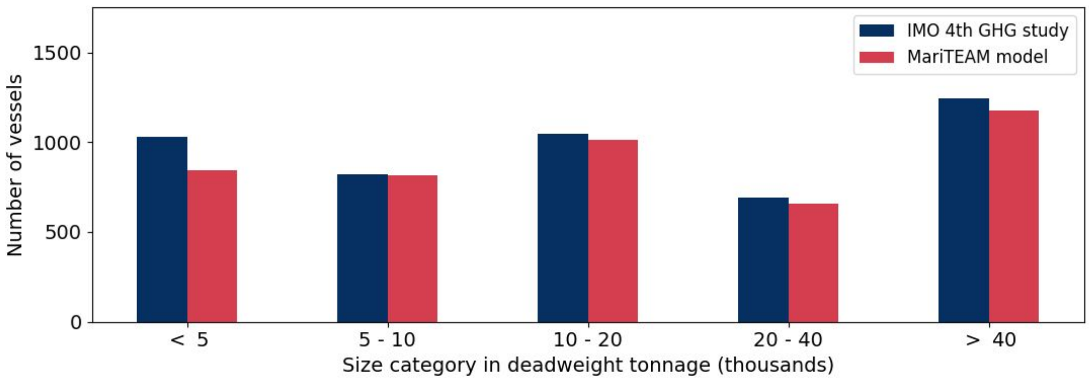

Figure S2. Comparison between number of chemical tanker vessels binned by deadweight tonnage evaluated in the 4th IMO GHG study and with the MariTEAM model in this study.

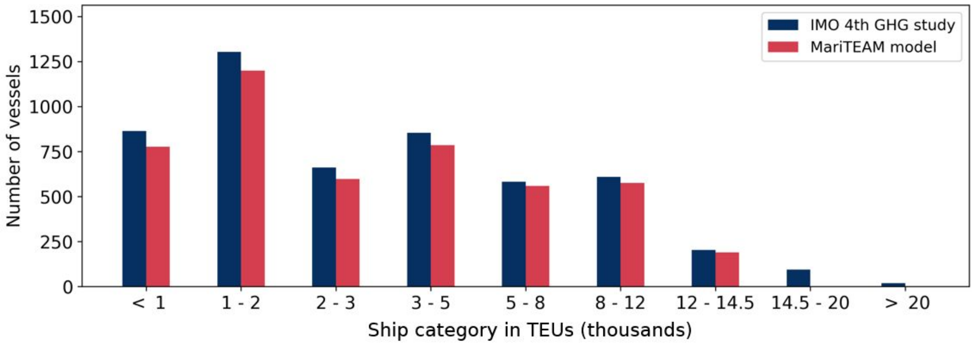

Figure S3. Comparison between number of container vessels binned by TEUs (twenty-foot equivalent unit) evaluated in the 4th IMO GHG study and with the MariTEAM model in this study.

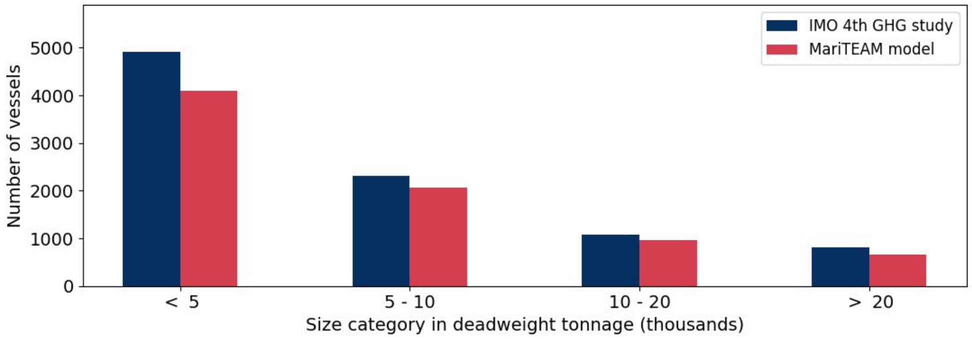

Figure S4. Comparison between number of general cargo vessels binned by deadweight tonnage evaluated in the 4th IMO GHG study and with the MariTEAM model in this study.

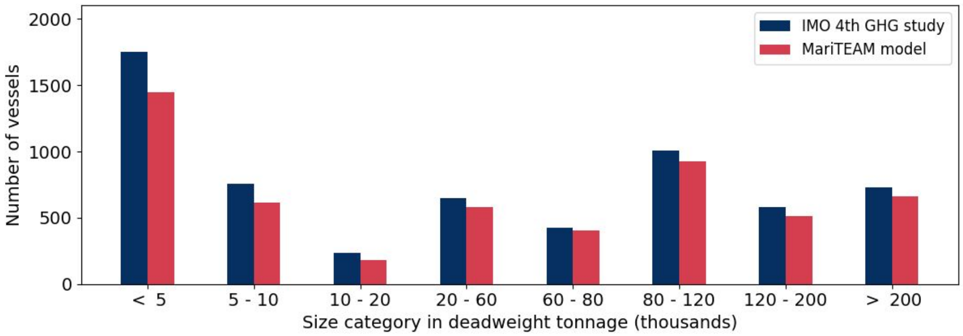

Figure S5. Comparison between number of oil tanker vessels binned by deadweight tonnage evaluated in the 4th IMO GHG study and with the MariTEAM model in this study.

Text S2. Ship voyage completer

Port callings registering date and time of arrival and departure from ports for each vessel were obtained from IHS Markit. This information was then applied to complete ship voyages, significantly improving route completions in regions where AIS data is scarce. The routes were

completed for shorter time intervals (0.1° latitude-longitude resolution) using the A* path-search algorithm (*1*) in combination with Dijkstra's algorithm (*2*). In total, 37% of AIS messages used in this study are generated by the algorithm, in particular for deep-sea regions.

A demonstration of this feature is shown in Figure S6 that displays an oil tanker voyage sailing between the Persian Gulf and the Mediterranean Sea, where the original AIS data (navy-blue points) are completed to generate a minimum resolution of 0.1 degrees of latitude and longitude.

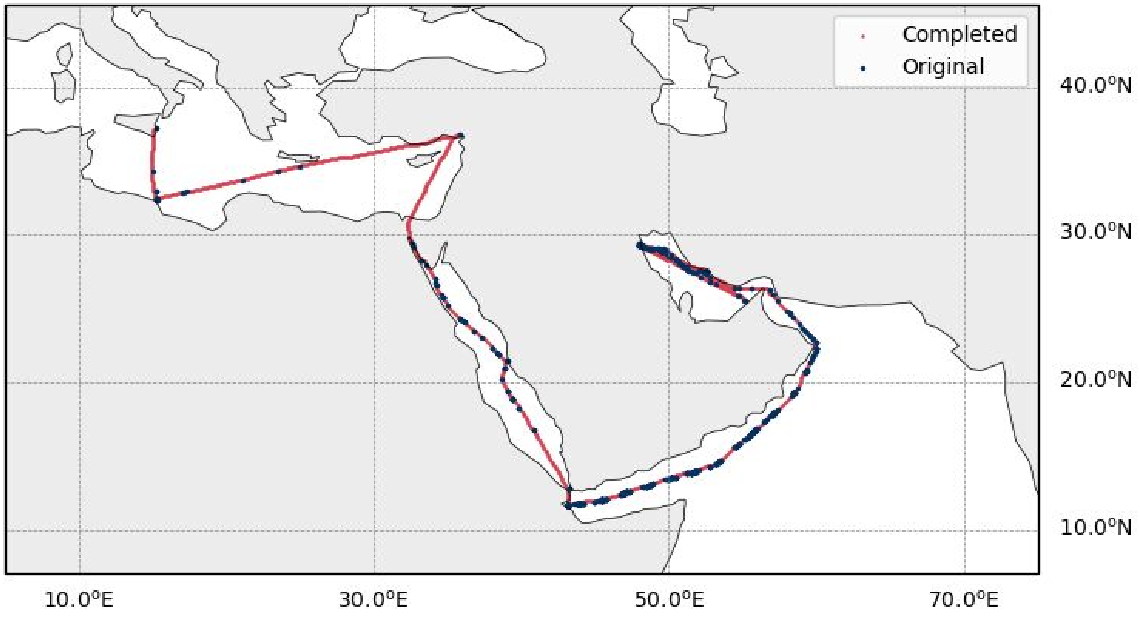

Figure S6. A ship voyage between the Persian Gulf and the Mediterranean completed with regular 0.1° points using the A* and Dijkstra algorithms.

Text S3. Power calculation

Total hull resistance (R_T) is essential to calculate ship's instantaneous power demand. For that, we calculate it as the combined result of the resistance in calm water (R_{cw}), the added resistance due to waves (R_{wave}) and due to wind (R_{wind}).

$$R_T = R_{cw} + R_{wave} + R_{wind} \quad (1)$$

The total resistance in calm water can be divided into three main components: frictional resistance (R_F), residual resistance (R_R), and air resistance (R_{AA}). The frictional component results from to tangential fluid forces and may account for 80% in lower speed cargo vessels and 50% for container carriers. Its formulation for a vessel with wetted surface (S) in a given speed (V) is shown in Equation 2, where the frictional coefficient (C_F) is given based on the ITTC-1957 model-ship correlation line. This component makes use of a form factor (k), which is implemented as a modification of Hollenbach's method using an empirical method proposed by MARINTEK (Equation 3).

$$R_F = C_F \frac{\rho V^2 S}{2} \quad (2)$$

For the residual resistance, which corresponds to the energy loss caused by waves created by the vessel, we combine the methods of Holtrop & Mennen (1982) and Hollenbach (1998). These are the most widely used methods for ship emission estimations (Nunes et al., 2017). The combination of the two methods is given through its average values. In addition, other components included are the resistance originating from the correlation allowance (R_A), bulbous bow effect (R_B), appendages (R_{APP}), and base drag (R_{BD}).

$$R_{cw} = (1 + k)R_F + R_R + R_{AA} + R_A + R_{APP} + R_B + R_{BD} \tag{3}$$

In order to assure that the values are sensible, the calm water resistance for each vessel is compared with the main engine's installed power, preventing unrealistic engine operational conditions. If engine load at design speed is below 80%, a factor is applied to scale up load to 85% at design speed. If load at design speed is above 95%, a factor is then applied to scale down the load to 90% at design speed. Hence engine use is not allowed to exceed installed engine size.

For added resistance, earlier studies (*4*,*5*,*6*) have made use of the method of (*3*). In the MariTEAM model, however, a different approach is taken. As the added resistance in waves may vary substantially across different ship segments, we adopt the added resistance calculation

proposed by ISO 15016:2015. The additional resistance is given as the sum of the additional resistance exerted by relative wind and waves.

For the component originating from relative wind, the method proposed applies the coefficient C_{AA} by combining the method of Blendermann (*7*) and the STAJIP (*8*) methods (Equation 4), depending on the ship segment analysed (*9*).

$$R_{wind} = 0.5\rho_A \cdot C_{AA}(\psi_{Wref}) \cdot A_{XV} \cdot V_{Wref}^2 - 0.5\rho_A \cdot C_{AA}(0) \cdot A_{XV} \cdot V_G^2 \qquad (4)$$

In which ρ_A is the air density, ψ_{Wref} is relative wind direction at reference height, A_{XV} is area of maximum transverse section exposed to the wind, V_{Wref} is the apparent wind speed at reference height and V_G is the ship speed over the ground.

For the added resistance due to waves we apply the STAWAVE-1 method shown in Equation 5 and complement it with STAWAVE-2 (*10*) method that approximate the transfer function of the mean resistance increase in heading regular waves.

$$R_{wave} = \frac{1}{16}\rho g H_{1/3}^2 B \sqrt{\frac{B}{L_{Bwl}}} \qquad (5)$$

Where B is the beam of the ship, $H_{1/3}$ is the significant wave height and L_{BWL} is length of the bow on the water line to 95% of maximum beam. The STAWAVE-2 method in shown in Equation 6, where $\overline{r_{aw}}(\omega)$ is the empirical transfer function.

$$R_{wave} = R_{AWML} + R_{AWRL} = 4\rho_s g(\zeta_a)^2 \frac{B^2}{L_{PP}} \overline{r_{aw}}(\omega) + \frac{1}{2}\rho_s g(\zeta_a)^2 B \alpha_1(\omega) \quad (6)$$

For applying these methods and model the weather effects on ship propulsion, we use historical reanalysis data for wind speed and direction, significant height of combined wind waves and swell, and mean wave direction.

Text S4. Propulsion Theory

To obtain the power delivered by the engine, we have to consider the losses that occur between the power effectively delivered and the power developed by the propeller. These effects are taken into account as the open-water efficiency (η_0), hull efficiency (η_H), relative rotative efficiency (η_R) and transmission efficiency (η_S).

The open-water efficiency (η_0) evaluates the performance of the propeller without the influence of the hull and is based on Wageningen-B series and the approximated method by (*11*). The

influence of the hull in the propeller inflow and the presence of the propeller itself is accounted by means of the hull efficiency (η_H), as shown in Equation 7.

$$\eta_H = \frac{1 - t}{1 - w} \qquad (7)$$

The thrust deduction factor (t) captures the increase in resistance caused by the propeller accelerating the water inflow whilst the wake factor (w) accounts for the speed difference between ship and the propeller inflow, both calculated as suggested by (*12*).

The relative rotative efficiency (η_R) accounts for the variable propeller blade loads due to the non-homogenous wake field inflow and normally ranges between 0.95 and 1.05 (*13*), being assumed 1.0 in the MariTEAM model. Last, the transmission efficiency (η_S) accounts for mechanical losses and losses in the transmission system due to shafts and bearings. We assume the direct drive efficiency of 0.97 suggested by (*13*).

Text S5. Effect of fouling on total hydrodynamic resistance

The model developed by (*14*) is applied to calculate the effect of fouling on vessels between docking periods. Ships are estimated to be docked for repair and maintenance every 5 years,

counting from the built year, when anti-fouling painting is re-applied on the hull surface. Average values for ship types are shown in Figure S7.

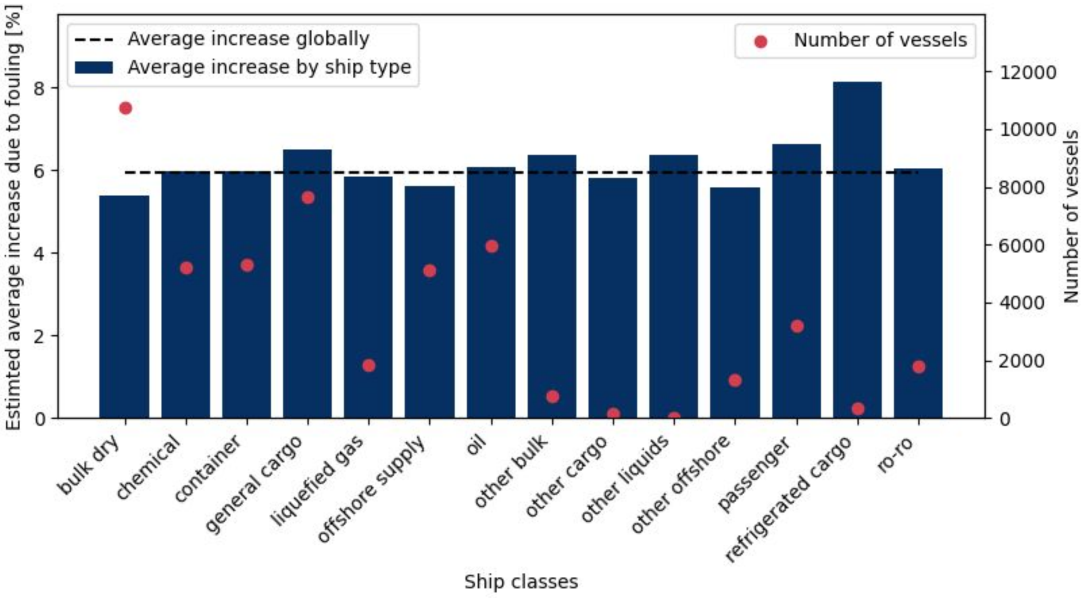

Figure S7. Average increase of total ship resistance due to fouling across different ship segments given the 2017 fleet.

Text S6. Auxiliary engines

The power calculation method presented serves to determine the operational load of the main engine. Auxiliary engines are modelled in another manner, as auxiliary engines characteristics are often not available in the ship database. For that, we follow the IMO guidelines for estimation installed power in auxiliary engines (MEPC 66/21, 2014). The method recommends the auxiliary

power demanded to be calculated as 2.5% of the main engine maximum continuous rating (MCR) plus 250 kW if MCR is higher than 10 MW, or as 5% of main engine if not. This method is applied regardless operational conditions (e.g., maneuvering, at port, berth). Specific factors depending on operation, ship class and capacity bin (e.g., IMO GHG studies) are expected to be included in future studies. The auxiliary engines are represented as a set of engines that can meet this demand, in which each of the engines is modeled as 1250 kW engines, 750 RPM, and 4-stroke, as most auxiliary engines present in the Sea-web Ships database are 4-stroke. In case of refrigerated cargo is transported, refrigerated container (or reefer) power needs are approximated to 4 kW per reefer, regardless of TEU (twenty-foot equivalent unit), which is an approximation based on the consumption range of 6 to 9 kW for a 40-foot container (*14*). Half the reefer points on a ship are considered to be in use on a typical voyage. Similarly, boilers are also accounted for, in special for containers for oil tankers, as supplementary power necessary at auxiliary engines, based on estimations presented by the 4th IMO GHG study (Faber et al., 2020).

Another source of emissions is the usage of installed scrubbers. Because this work focuses on analysing the global fleet for the year 2017, in which only 388 vessels were estimated to have this device installed (*15*), the effect of scrubbers has not been considered.

Text S7. Power calculations compared with in-service data

The power estimations developed in this model have been compared with in-service shaft power in order to understand the degree of uncertainty. Figure S8 presents one such example for an LNG carrier with overall length of ~285 meters, DWT of ~96000 tonnes, and installed power of ~24 MW. The results demonstrate the capabilities of the model to capture modifications on the operation as well as the sea state conditions with a 6% average difference for the evaluated period.

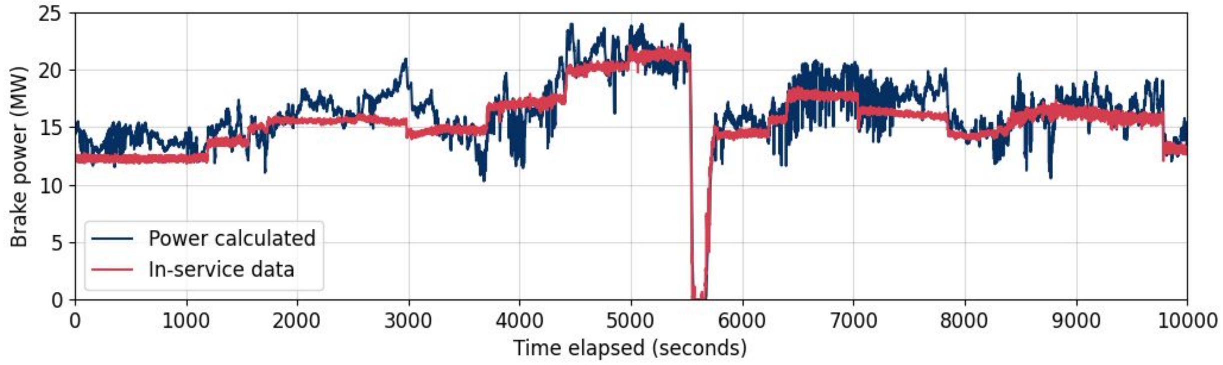

Figure S8. A time series comparison between the MariTEAM model (coral line) brake power (MW) calculation and in-service data from an LNG tanker (navy-blue line).

Text S8. Ship emissions

Emissions are the result of the combustion inside the engine chamber and are directly dependent of the engine configuration and fuel properties. Whereas carbon dioxide (CO_2) and water are originated as result of the hydrocarbon structure of fossil fuels, other emissions occur due to non-ideal circumstances during combustion, such as incomplete combustion of fuel due to inadequate air/fuel mixture (CO), insufficient temperature near the cylinder wall (hydrocarbons), combustion of engine lubricating oil (OC), presence of non-hydrocarbon components in the fuels (SO_X), temperatures above 1600°C in the cylinders (NO_X), unburned fuel (PM), among other causes (*16*).

Engines are considered to be operating in ideal conditions and maintenance has been done accordingly. This is, naturally, a simplification and difficult to be thoroughly investigated. Nevertheless, increase in emissions due to poor maintenance should be accounted for in future studies. For instance, NO_X emissions might increase more than 7 g kWh^{-1} due to fault in advanced injection timing. Fault in the nozzle may increase CO emissions by 1.65 g kg^{-1} fuel at full loading (*17*).

Fuel consumption is calculated based on the load conditions at the engine and the specific fuel consumption (*SFOC*). Whenever operating outside its optimal range, fuel consumption will

increase accordingly, reaching up to 62% increase. The fuel consumption is then multiplied by the content of carbon or sulphur present (*FC*) in the fuel to obtain CO_2 and SO_X emissions (*18*), as shown in Equation 8.

$$E_{i,j,k} = \sum_j P_{Sj,k} \cdot SFOC_{i,j} \cdot FC_{j,k} \cdot \Delta t_k \qquad (8)$$

Sulphur content is approximated to 2.7% in mass for HFO and 0.1% for MGO. Sulphur oxides are split in SO_2 and SO_4 by 97% and 3% respectively.

Emissions of other pollutants that are not strictly related to the fuel chemical composition, but rather vary depending on optimal combustion conditions are calculated differently. In those cases, shaft power (P_S) is multiplied by emission factors (*EF*) that are corrected based on engine load as a percentage of MCR (maximum continuous rating) (*LF*) and are modelled as polynomial or power equations obtained through regression models based on a literature review.

$$E_{i,j,k} = \sum_j P_{S_{j,k}} \cdot EF_{i,j} \cdot LF_{j,k} \cdot \Delta t_k \qquad (6)$$

Here, *i* is the pollutant being assessed, *j* is the engine (main or auxiliary engine), *k* is the route section. The exception is the case of NO_X emissions, they are stipulated as the maximum allowed according to IMO in that region, respecting the engine RPM and tier, resulting in emission rates

between 2.96 and 17 g kWh^{-1}. For black carbon, we use the approximations developed by (*19*), that establish regression curves based on the study of several engines.

These emission factors modelled in our study are shown in Figure S9, where the boxes contain the emission values during nearly optimal conditions (60-95% MCR) and the whiskers show the lower and upper quartile, while scatter points denotate the maximum and minimum values that could be achieve in other conditions. Because aerosols are highly affected by sub-optimal operational conditions, emission factors can tenfold in some cases (Figure S9b).

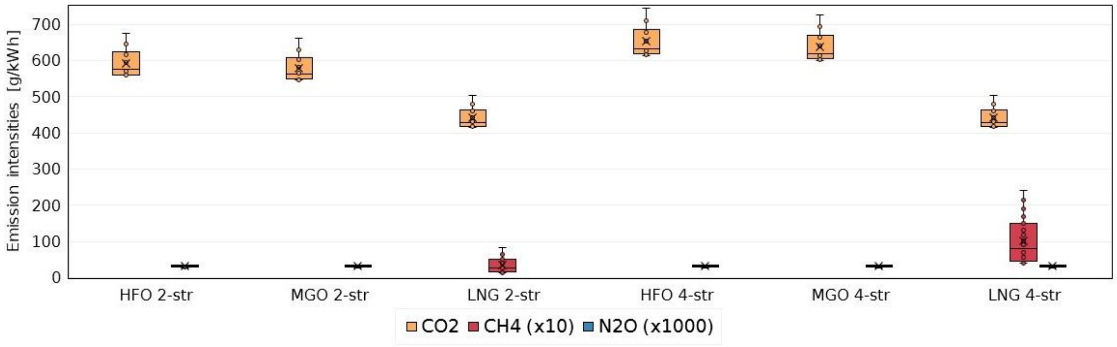

(a)

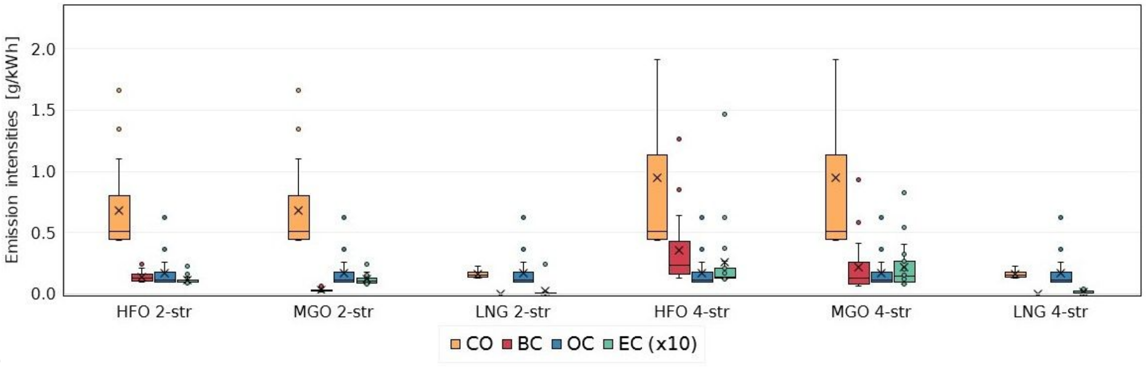

(b)

Figure S9. Emission factors (g kWh^{-1}) range between 10-100% MCR for GHGs (a), i.e. CO_2, CH_4 and N_2O, and aerosols (b), i.e. CO, OC, EC, BC, for engines fueled by HFO, MGO and LNG with 2 and 4 strokes.

Text S9. Fuel production

Figure S10 shows a Material flow analysis (MFA) for emissions and losses in fuel production applied in our study in an energy-based Sankey diagram. In total, nearly 13% of hydrocarbon extracted are lost in the process of extracting, producing, and processing as direct emissions or material discharge.

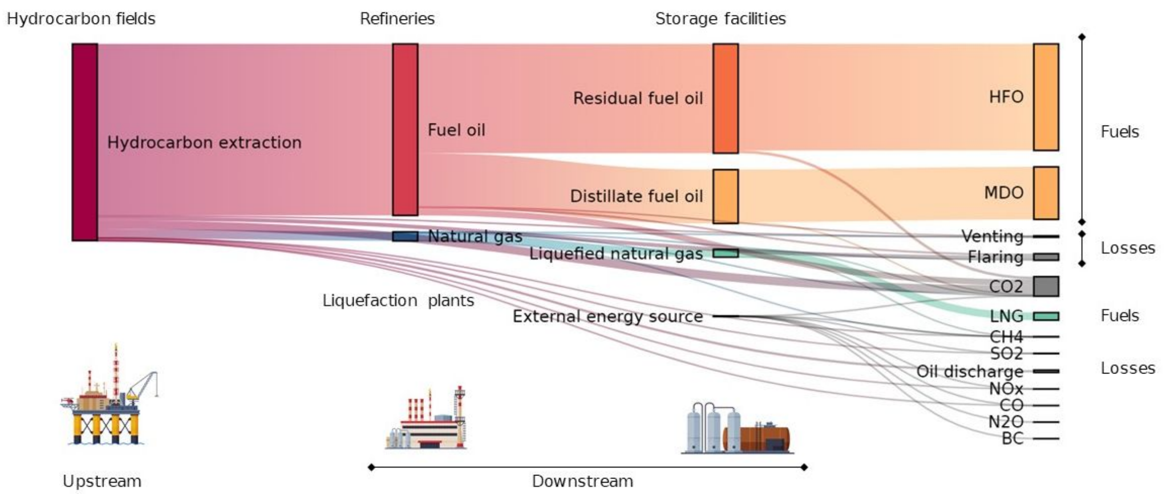

Figure S10. Material flow analysis for one unit of hydrocarbon extracted to produce HFO, MGO and LNG based on shipping fuel mix for the year 2017, including air emissions and material losses, i.e. venting, flaring, material discharge.

Text S10. Global shipping emissions by pollutant

The spatial distribution of other pollutants, in addition to CO_2 emissions presented in the main text, are shown in Figures S11 to S20 and include, respectively, CH_4, N_2O, NMVOC, NO_X, SO_X, CO, OC, EC, and BC emissions.

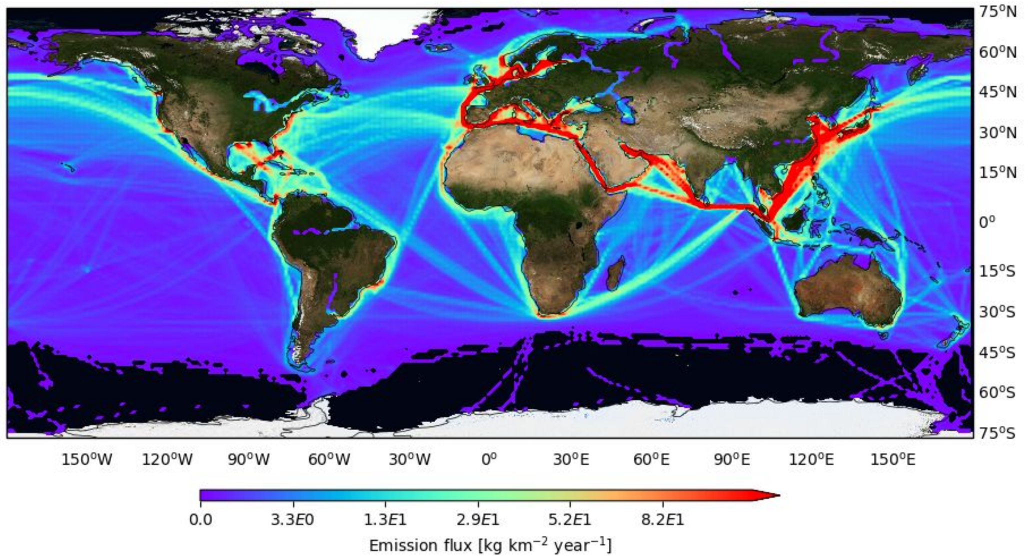

Figure S11. Geospatial distribution of CH_4 emissions (kg m^{-2} s^{-1}) for tank-to-wake global shipping, totalling 107 thousand tonnes in the year 2017.

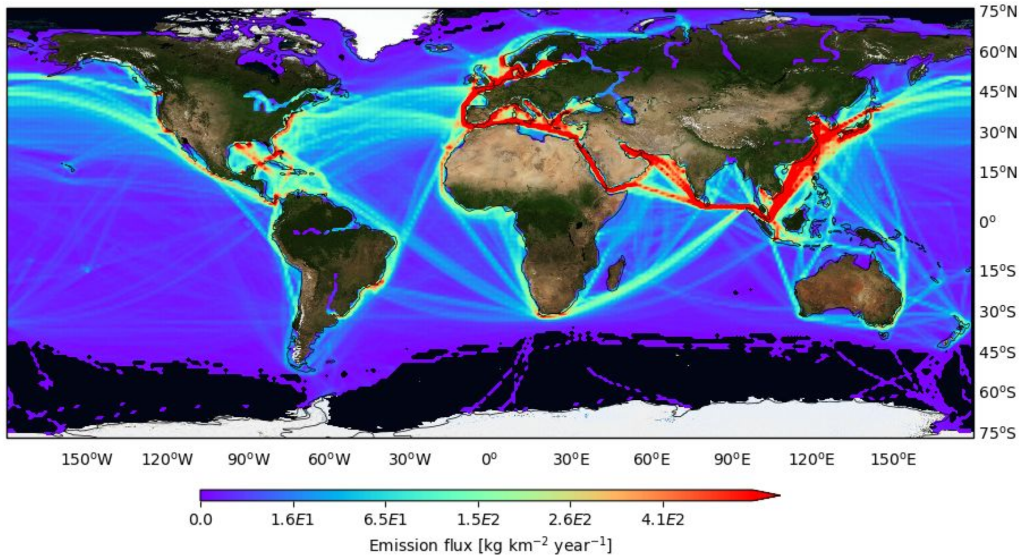

Figure S12. Geospatial distribution of N_2O emissions (kg m^{-2} s^{-1}) for tank-to-wake global shipping, totalling 50 thousand tonnes in the year 2017.

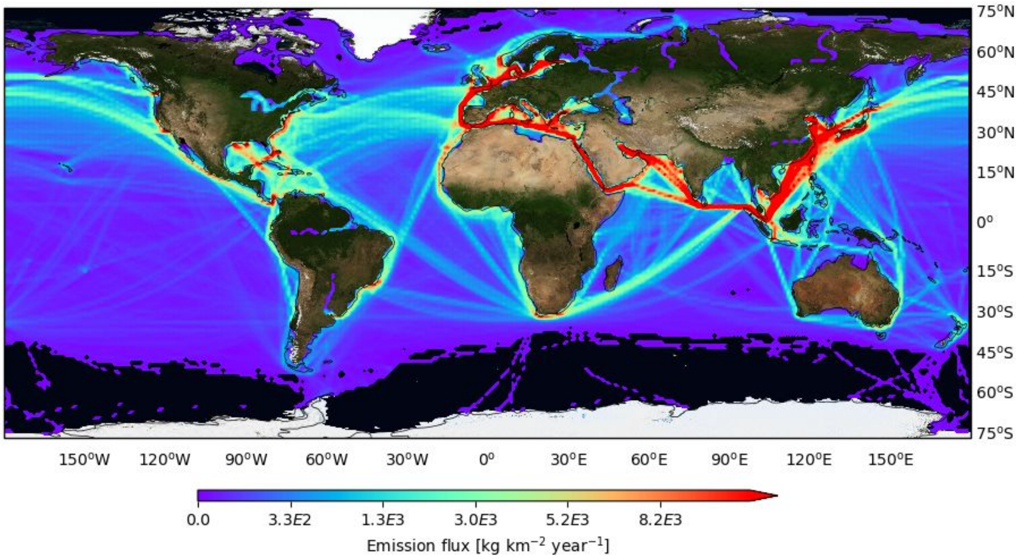

Figure S13. Geospatial distribution of NMVOC emissions (kg m^{-2} s^{-1}) for tank-to-wake global shipping, totalling 1001 thousand tonnes in the year 2017.

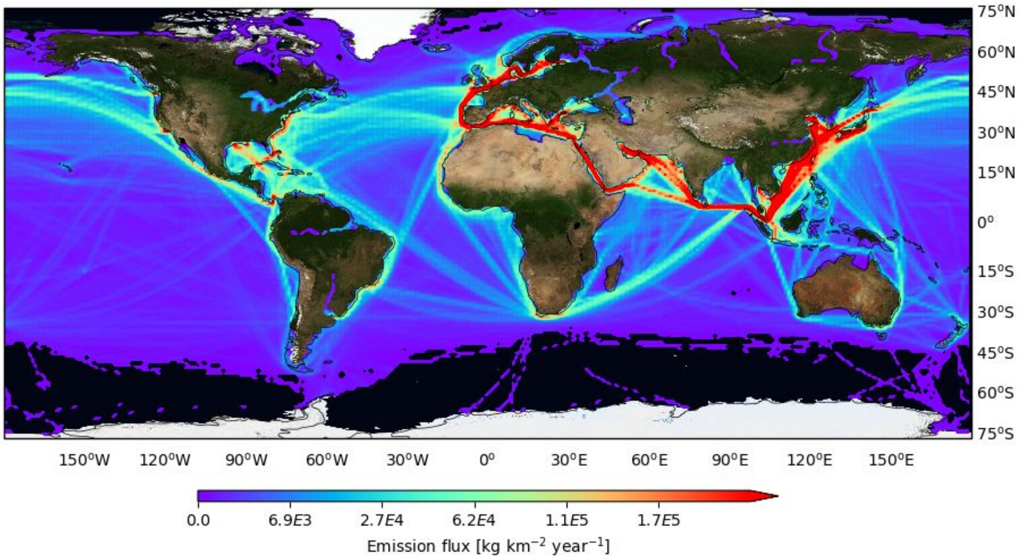

Figure S14. Geospatial distribution of NO_X emissions (kg m^{-2} s^{-1}) for tank-to-wake global shipping, totalling 18.6 million tonnes in the year 2017.

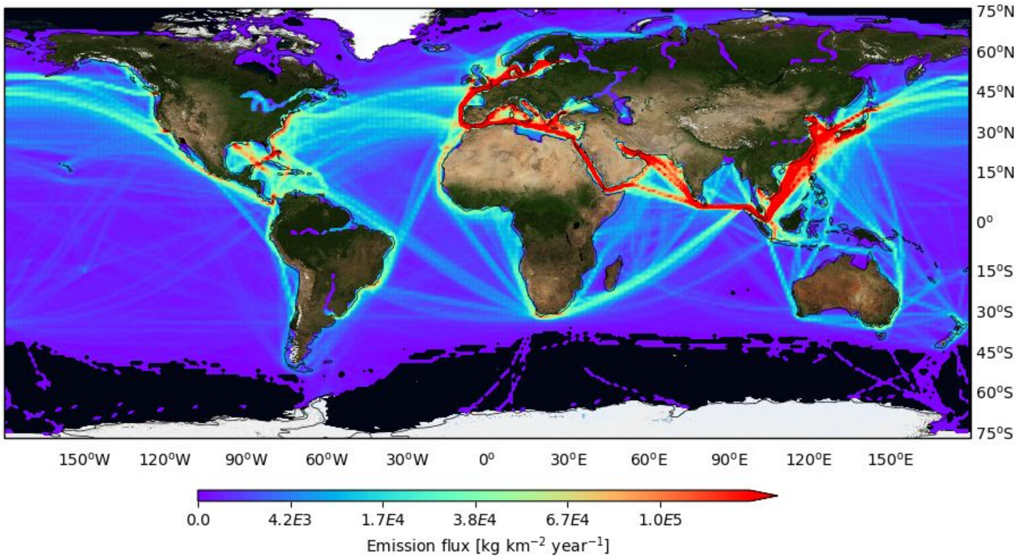

Figure S15. Geospatial distribution of SO_2 emissions (kg m^{-2} s^{-1}) for tank-to-wake global shipping, totalling 9 million tonnes in the year 2017.

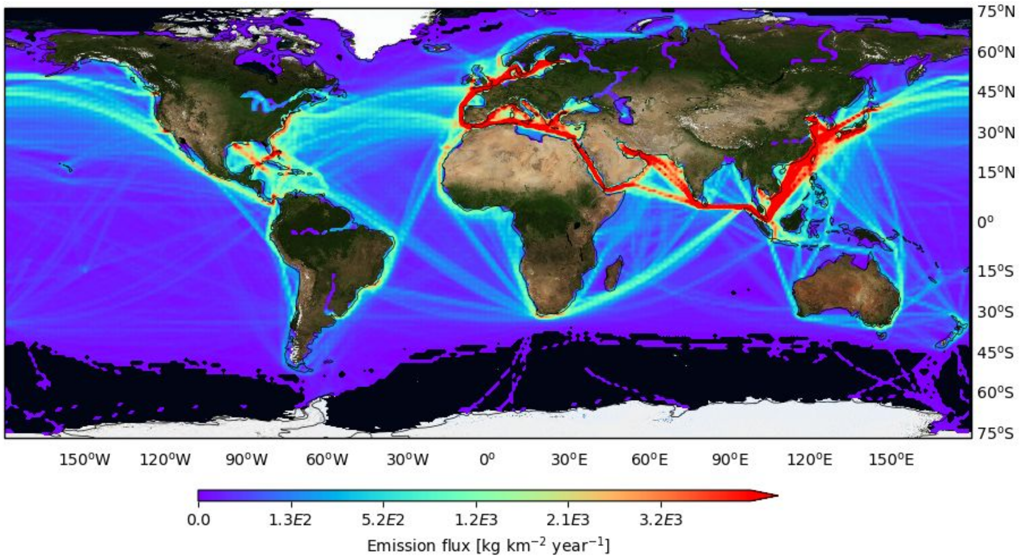

Figure S16. Geospatial distribution of SO_4 emissions (kg m^{-2} s^{-1}) for tank-to-wake global shipping, totalling 225 thousand tonnes in the year 2017.

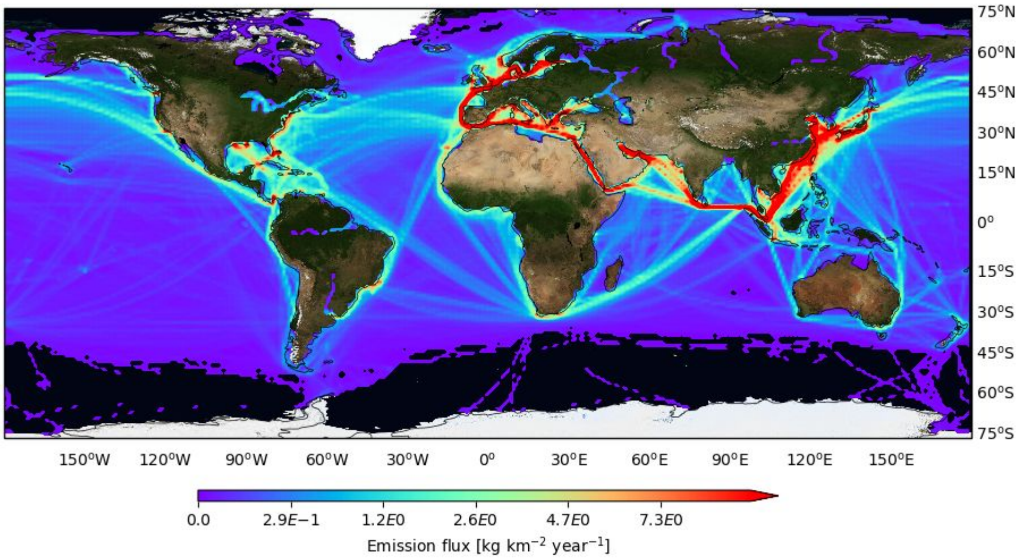

Figure S17. Geospatial distribution of CO emissions (kg m^{-2} s^{-1}) for tank-to-wake global shipping, totalling 642 thousand tonnes in the year 2017.

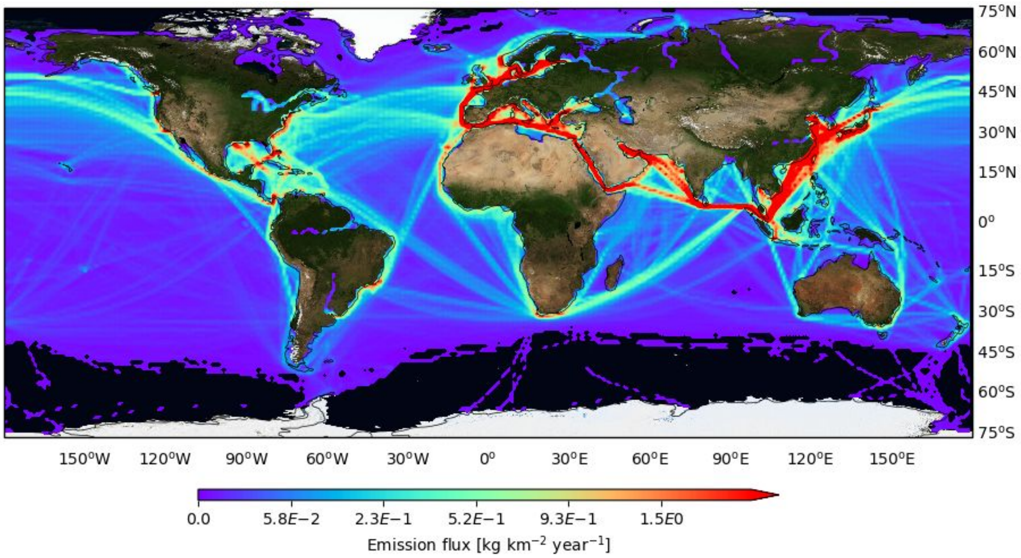

Figure S18. Geospatial distribution of organic carbon (OC) emissions (kg m^{-2} s^{-1}) for tank-to-wake global shipping, totalling 173 thousand tonnes in the year 2017.

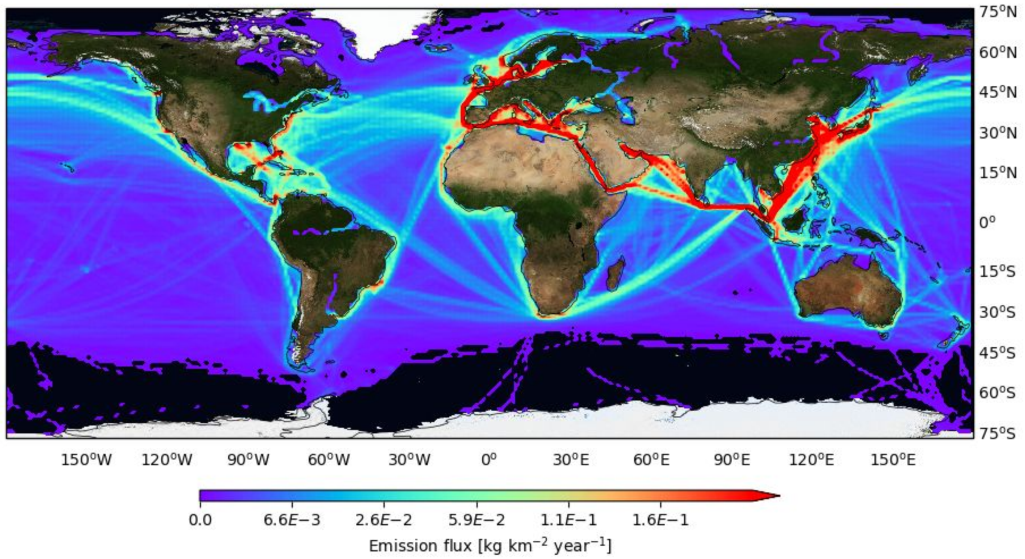

Figure S19. Geospatial distribution of elementary carbon (EC) emissions (kg m^{-2} s^{-1}) for tank-to-wake global shipping, totalling 15 thousand tonnes in the year 2017.

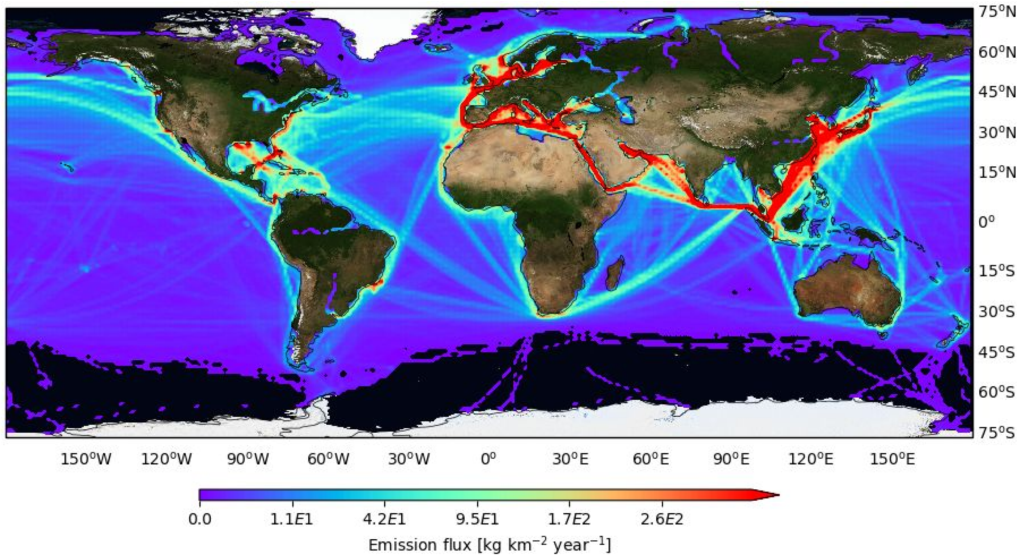

Figure S20. Geospatial distribution of black carbon (BC) emissions (kg m^{-2} s^{-1}) for tank-to-wake global shipping, totalling 25 thousand tonnes in the year 2017.

Text S11. Fuel production supply for global shipping emissions by pollutant

In addition to ship emissions, we also present the spatial distribution of fuel production emissions for CH_4, N_2O, NMVOC, NO_X, SO_X, CO, and BC emissions in Figures S21 to S27. Note that the scale differs between the figures.

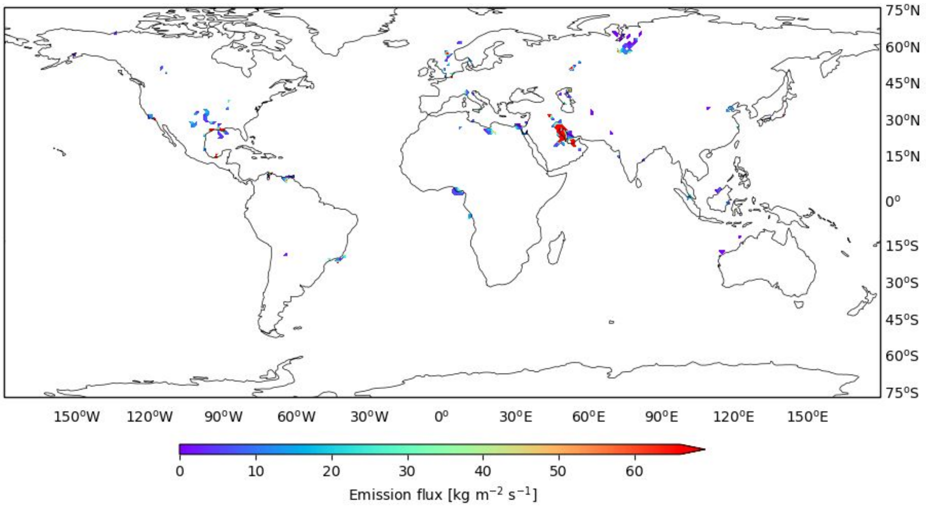

Figure S21. Geospatial distribution of methane (CH_4) emissions (kg m^{-2} s^{-1}) for well-to-tank global shipping, totalling 12 thousand tonnes in the year 2017.

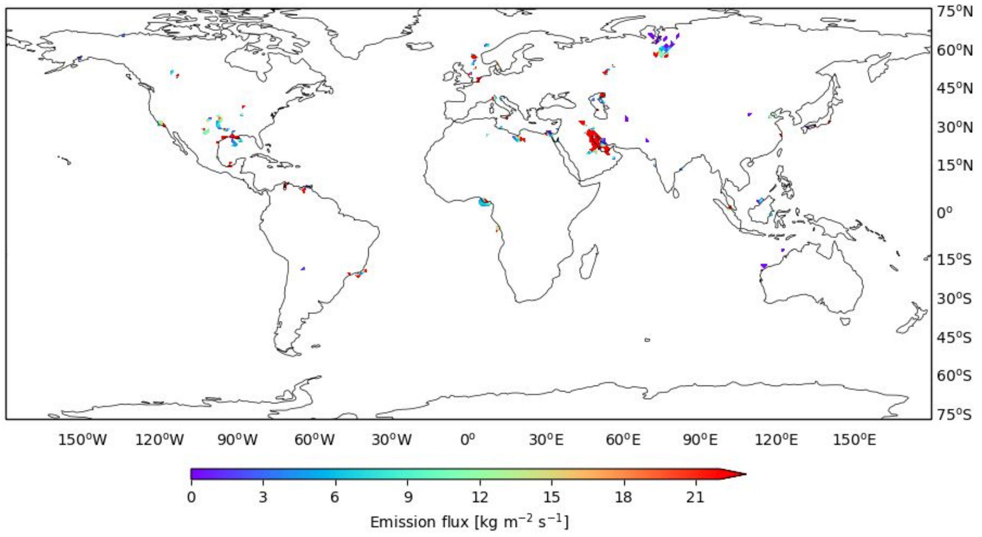

Figure S22. Geospatial distribution of N_2O emissions (kg m^{-2} s^{-1}) for well-to-tank global shipping, totalling 3.9 thousand tonnes in the year 2017.

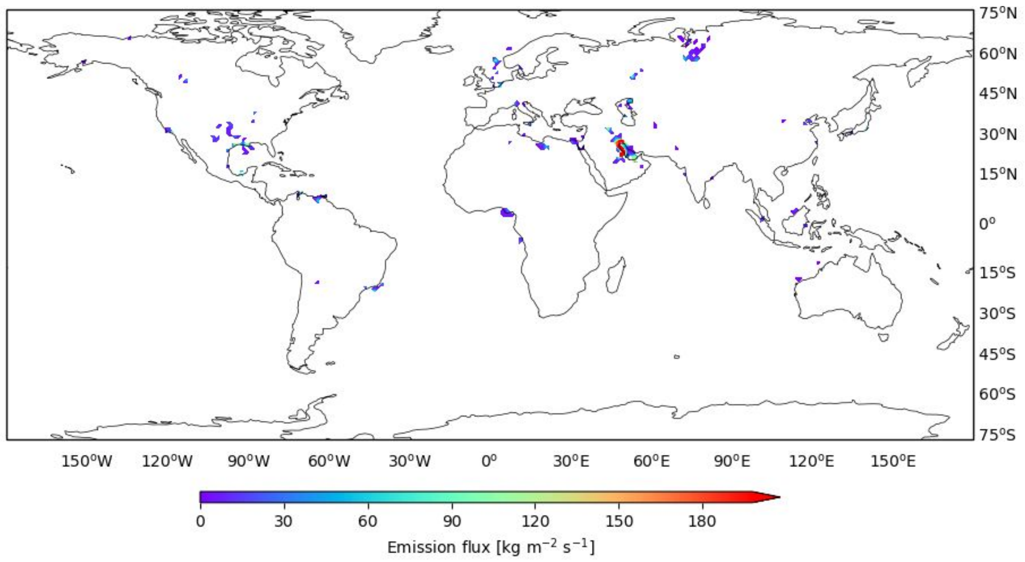

Figure S23. Geospatial distribution of NMVOC emissions (kg m^{-2} s^{-1}) for well-to-tank global shipping, totalling 42 thousand tonnes in the year 2017.

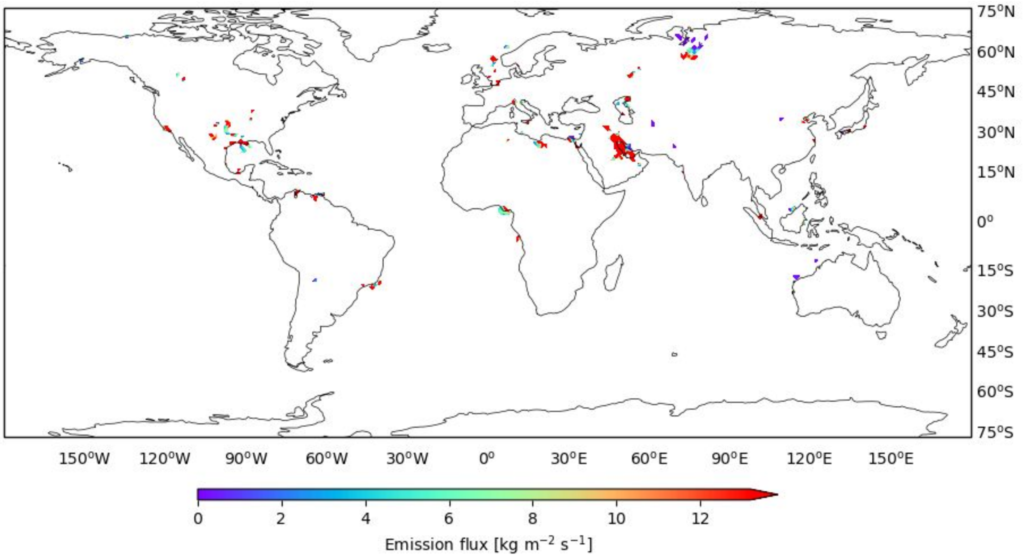

Figure S24. Geospatial distribution of NO_X emissions (kg m^{-2} s^{-1}) for well-to-tank global shipping, totalling 2.2 million tonnes in the year 2017.

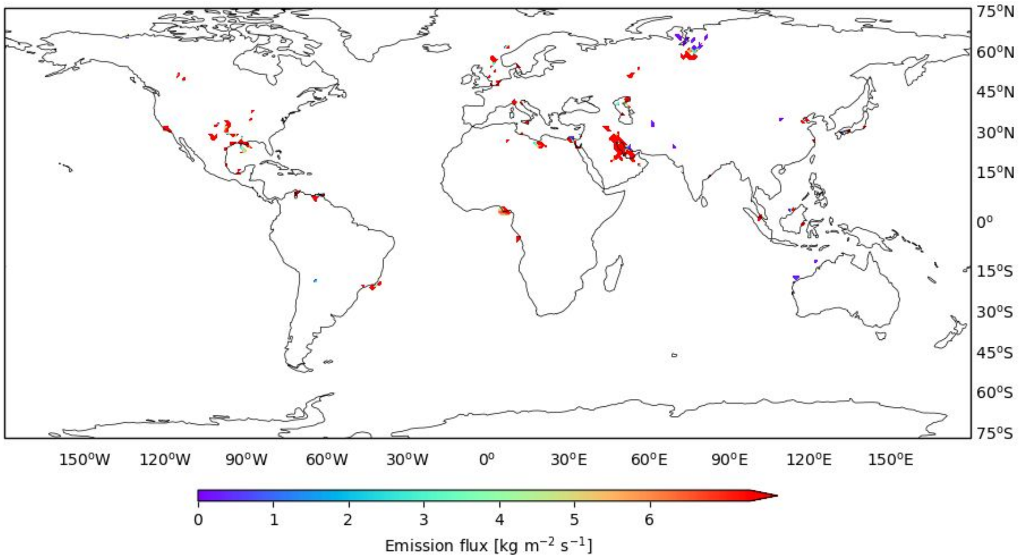

Figure S25. Geospatial distribution of SO_X emissions (kg m^{-2} s^{-1}) for well-to-tank global shipping, totalling 1.2 million tonnes in the year 2017.

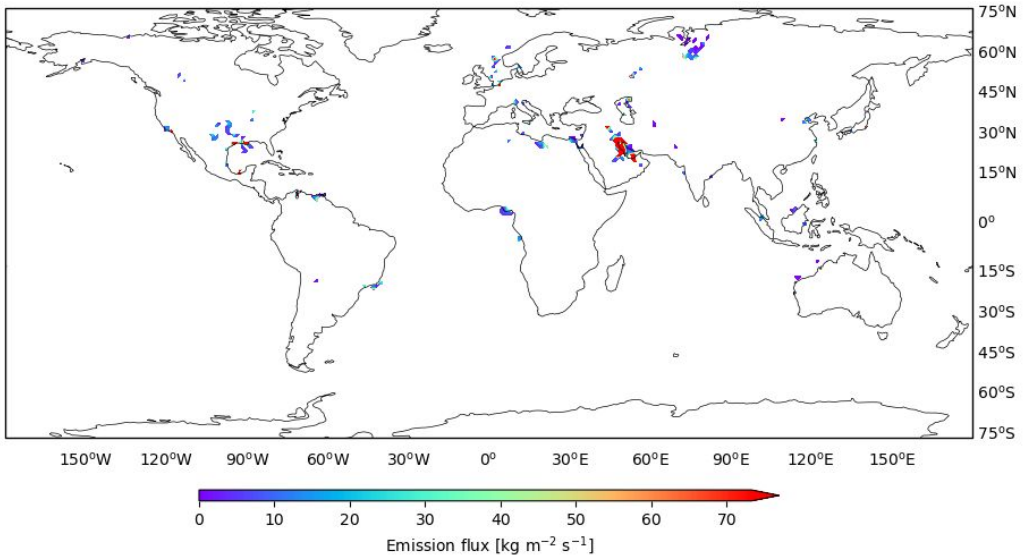

Figure S26. Geospatial distribution of carbon monoxide (CO) emissions (kg m^{-2} s^{-1}) for well-to-tank global shipping, totalling 15 thousand tonnes in the year 2017.

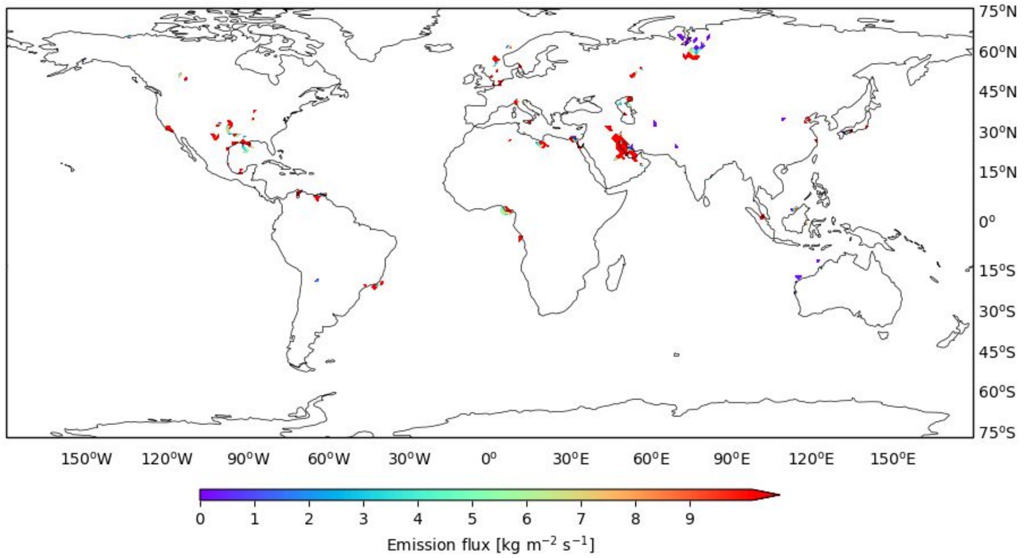

Figure S27. Geospatial distribution of black carbon (BC) emissions (kg m^{-2} s^{-1}) for well-to-tank global shipping, totalling 2.1 thousand tonnes in the year 2017.

Text S12. Emissions aggregated by ship type

The difference between resistance-power models, such as the ones applied in this study, and the load-factor models that adapt the Admiralty formula for a reference power at given speed and draft is investigate in Figure S28 for the global fleet with a sea margin of 12.5%. The spatial distribution of estimated instantaneous power indicate that load-based models result in higher values (~10%) for lower latitudes or sheltered waters where the effect of weather is less significant, in contrast to lower values in higher latitudes due to the effect of added resistance to waves and wind.

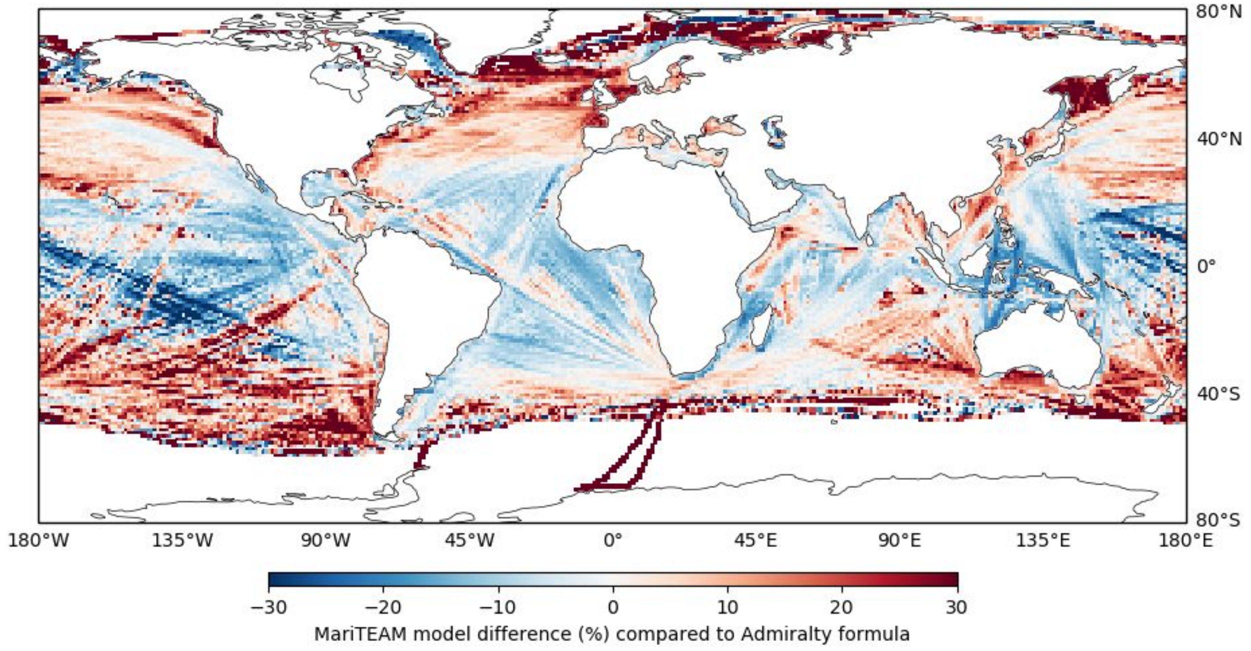

Figure S28. Comparison for instantaneous power estimating between the MariTEAM model and the Admiralty formula using Holtrop & Mennen method as reference power plus 12.5% sea margin.

Text S13. Emissions aggregated by ship type

Ship segments contribute to total amount of emissions in different proportions. Figure S29, for instance, illustrates the contribution to CO_2 emissions. Moreover, emissions are not entirely proportional to the ship segment, in special due to the operational conditions that each segment is subject to. For container speeds (11% of global fleet), higher service speeds lead to a bigger share in emissions (31%). For offshore supply vessels (10% of global fleet), short and fragmented operations lead to a significantly smaller contribution to CO_2 emission (1%).

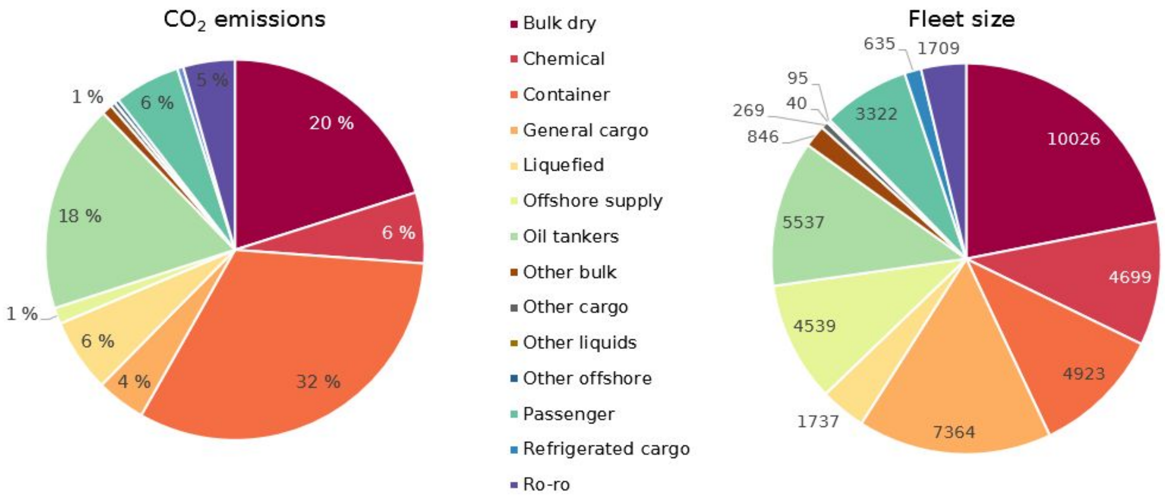

Figure S29. Ship CO_2 emissions contribution by ship type and comparison with its correspondent size in terms of number of vessels.

References

1. Hart, P. E.; Nilsson, N. J.; Raphael, B. A formal basis for the heuristic determination of minimum cost paths. *IEEE transactions on Systems Science and Cybernetics* **1968,** *4* (2), 100-107.

2. Dijkstra, E. W. A note on two problems in connexion with graphs. *Numerische mathematik* **1959,** *1* (1), 269-271.

3. Kwon, Y. J. Speed loss due to added resistance in wind. *Naval Architecture* **2008,** *3,* 14-16.

4. Lu, R.; Turan, O.; Boulougouris, E.; Banks, C.; Incecik, A. A semi-empirical ship operational performance prediction model for voyage optimization towards energy efficient shipping. *Ocean Engineering* **2015,** *110,* 18--28.

5. Kim, M.; Hizir, O.; Turan, O.; Day, S.; Incecik, A. Estimation of added resistance and ship speed loss in a seaway. *Ocean Engineering* **2017,** *141,* 465--476.

6. Mersin, K. A New Method for Calculating Fuel Consumption by Using Speed Loss Function. *International Journal of Environment and Geoinformatics* **2021,** *7* (1), 64--67.

7. Blendermann, W. Parameter identification of wind loads on ships. *Journal of Wind Engineering and Industrial Aerodynamics* **1994,** *51* (3), 339-351.

8. Boom, H. v. d.; Huisman, H.; Mennen, F. *New Guidelines for Speed/Power Trials;* SWZ/Maritime, 2013.

9. Dale, T. *Development of simplified methods for ship powering performance calculations;* NTNU: Trondheim, 2020.

10. ITTC. *Analysis of Speed and power trial data;*, 2015.

11. Andersen, P. *Hydrodynamics of ship propellers;* Cambridge University Press, 1994.

12. Harvald, S. A. Resistance and propulsion of ships. **1992**.

13. Kristensen, H. O.; Lützen, M. Prediction of resistance and propulsion power of ships. *Clean Shipping Currents* **2012,** *1* (6), 1-52.

14. Filina-Dawidowicz, L.; Filin, S. Innovative energy-saving technology in refrigerated containers transportation. *Energy Efficiency* **2019,** *12* (5), 1151-1165.

15. Comer, B.; Georgeff, E.; Osipova, L. *Air emissions and water pollution discharges from ships with scrubbers;* ICCT, 2020.

16. Recitoglu, I. A.; Altinisik, K.; Keskin, A. The pollutant emissions from diesel-engine vehicles and exhaust aftertreatment systems. *Clean Technologies and Environmental Policy* **2015,** *17* (1), 15-27.

17. Duran, V.; Uriondo, Z.; Moreno-Gutiérrez, J. The impact of marine engine operation and maintenance on emissions. *Transportation Research Part D: Transport and Environment* **2012,** *17* (1), 54-60.

18. Eggleston, S.; Buendia, L.; Miwa, K.; Ngara, T.; Tanabe, K. *2006 IPCC guidelines for national greenhouse gas inventories;* Institute for Global Environmental Strategies Hayama, Japan, 2006.

19. Olmer, N.; Comer, B.; Roy, B.; Mao, X.; Rutherford, D. *Greenhouse gas emissions from global shipping, 2013-2015 Detailed methodology;* ICCT (The International Council on Clean Transportation), 2017.

20. Holtrop, J.; Mennen, G. G. J. An approximate power prediction method. *International Shipbuilding Progress* **1982,** *29* (335), 166-170.

21. Hollenbach, K. U. Estimating resistance and propulsion for single-screw and twin-screw ships. *Schiffstechnik* **1998,** *45* (2), 72.

22. Nunes, R. A. O.; Alvim-Ferraz, M. C. M.; Martins, F. G.; Sousa, S. I. V. The activity-based methodology to assess ship emissions - A review. *Environmental Pollution* **2017,** *231* (x), 87-103.
